# Optimization of Biomimetic, Leukocyte-Mimicking Nanovesicles for Drug Delivery Against Colorectal Cancer Using a Design of Experiment Approach

**DOI:** 10.3389/fbioe.2022.883034

**Published:** 2022-06-08

**Authors:** Riccardo Rampado, Andrea Biccari, Edoardo D’Angelo, Federica Collino, Giulia Cricrì, Paolo Caliceti, Federica Giordano, Francesca Taraballi, Salvatore Pucciarelli, Marco Agostini

**Affiliations:** ^1^ Department of Surgical, Oncological and Gastroenterological Sciences, University of Padua, Padua, Italy; ^2^ Nano-Inspired Biomedicine Lab, Institute of Pediatric Research- Città della Speranza, Padua, Italy; ^3^ Department of Clinical Sciences and Community Health, University of Milano, Milan, Italy; ^4^ Laboratory of Translational Research in Pediatric Nephro-Urology, Fondazione Ca’ Granda IRCCS Ospedale Maggiore Policlinico, Milan, Italy; ^5^ Department of Pharmaceutical and Pharmacological Sciences, University of Padua, Padua, Italy; ^6^ Center for Musculoskeletal Regeneration, Houston Methodist Academic Institute, Houston, TX, United States; ^7^ Orthopedics and Sports Medicine, Houston Methodist Hospital, Houston, TX, United States

**Keywords:** biomimetic, nanomedicine, inflammation, design of experiment, drug delivery, colorectal cancer

## Abstract

The development of biomimetic nanoparticles (NPs) has revolutionized the concept of nanomedicine by offering a completely new set of biocompatible materials to formulate innovative drug delivery systems capable of imitating the behavior of cells. Specifically, the use of leukocyte-derived membrane proteins to functionalize nanovesicles (leukosomes) can enable their long circulation and target the inflamed endothelium present in many inflammatory pathologies and tumors, making them a promising and versatile drug delivery system. However, these studies did not elucidate the critical experimental parameters involved in leukosomes formulation. In the present study, we approached the preparation of leukosomes using a design of experiment (DoE) method to better understand the influence of experimental parameters on leukosomes features such as size, size distribution, and protein loading. We also validated this formulation technologically and tested its behavior in *in vitro* colorectal cancer (CRC) models, including CRC patient-derived tumor organoids (PDOs). We demonstrated leukosomes biocompatibility, endothelium adhesion capability, and tumor target in three-dimensional (3D) settings using CRC cell lines. Overall, our study offers a novel conceptual framework for biomimetic NPs using a DoE strategy and consolidates the high therapeutic potential of leukosomes as a viable drug delivery system for anti-inflammatory and antineoplastic applications.

## 1 Introduction

The optimization of pharmaceutical formulations may represent a daunting challenge since even relatively simple processes can be affected by many variables, each one with its own potential contribution to the results. The need to control all these variables often leads to time-consuming and very expensive screening studies that require the analysis of a single variable at a time. Thus, a much more straightforward trial and error process is often employed. This strategy is cheaper and more time-efficient, but it does not provide much insight into the contribution of different experimental parameters to the experimental outcome since its design is focused only on the result. Furthermore, the final solutions found through trial and error are not necessarily the best possible ones since the scope of the study is quite narrow by its own nature. On the other hand, only very large designs allow an understanding of the potential interplay among the experimental parameters involved (Politis et al., 2017). The design of experiment (DoE) strategy can overcome these limitations. DoE is a mathematical approach that enables the creation of specific combinations of qualitative and quantitative experimental parameters under investigation, creating an “experimental space” in which each dimension is represented by one of the experimental parameters. These combinations are calculated within a specified range of parametric values and are arranged in a pattern that allows obtaining the maximum amount of information on the process while performing the minimal number of experimental runs (Politis et al., 2017). The selection of which DoE pattern should be employed for an investigation is a critical choice and depends on the structure of the experiment, the number of experimental parameters that are included, and the intended result (Tavares Luiz et al., 2021). Specifically, DoE can be used to understand which among the selected parameters is statistically relevant in determining the results of the process (screening objective); create mathematical models that allow to predict the results based on the specific values of the selected parameters (response surface method objective); troubleshoot, and optimize or make more robust a specific process (Weissamn and Anferson, 2015).

In the last years, the advent of biomimetic nanovectors revolutionized the field of nanomedicine by introducing a completely new paradigm of design for nanovectors. The biomimetic strategy is based on the use of cell membrane and membrane proteins to formulate vectors that can recapitulate the behavior of the cells they are derived from (Sushnitha et al., 2020; Zhang et al., 2020). This strategy employs highly biocompatible building blocks to create sophisticated formulations with a variety of potential effects. Nevertheless, most studies to this day are still focused on the strict evaluation of biomimetic nanovectors, and not much attention is paid to the optimization and understanding of their formulative process. Among these, leukosomes (Leukos) are biomimetic nanovesicles that are functionalized on their surface with membrane proteins derived from leukocytes with the aim to induce longer Leukos circulation thanks to the presence of “stealth” inducing proteins (CD45 and CD47) and actively target them to activated endothelia associated with inflammatory diseases and tumors through adhesion proteins (CD-11b, PSGL-1, and integrins) (Molinaro et al. 2016, E. M. Molinaro R, Design and Development of Biomimetic Nanovesicles Using a Microfluidic Approach. 2018, Molinaro et al., 2020; Martinez et al., 2018; Zinger et al., 2021; Zinger et al., 2020; Corbo et al., 2017).

In this project, a single DoE was employed to understand which variables are relevant in influencing the features of Leukos using a microfluidic approach. Furthermore, DoE was used to optimize the process itself *via* the calculation of parameters that would yield desirable NPs features. This strategy has already been applied successfully to liposomal formulations (Sedighi et al., 2019). The parameters were validated by a deep characterization of Leukos physiochemical features. We also tested the possibility to load this formulation with the antitumor drug doxorubicin (DOXO) and to label them with Cyanine 5.5 (Cy5.5) for theranostic purposes. We assessed Leukos uptake by and cytotoxic effect on colorectal cancer (CRC) cell line and patient-derived CRC organoids (PDO) and their uptake from inflamed endothelial cells and macrophages. We believe that this study may work as a proof of concept for the creation of new formulation frameworks for other biomimetic nanovectors with a variety of diagnostic and therapeutic applications using the DoE approach.

## 2 Materials and Methods

### 2.1 Materials and Cell Cultures

HCT-116 epithelial CRC cells (CCL-247) were purchased from ATCC and cultured in T25 cell culture flasks using high glucose Dulbecco’s modified Eagle’s Medium (DMEM, Millipore Sigma, D5671-500ML) supplemented with 10% Fetal Bovine Serum (FBS, Atlas Biologicals, F-0500-A) and with 1% Pen/Strep Solution [Gibco™ Penicillin-Streptomycin (10,000 U/ml), 15140122]. Human leukemia-derived, stabilized monocytes (THP-1, ATCC^®^ TIB-202™) were purchased from ATCC. THP-1 cells were cultured in T175 cell culture flasks (Thermo Scientific™ Nunc™ EasYFlask™ Cell Culture Flasks, 159910) using RPMI-1640 medium (ATCC^®^ 30-2001™) supplemented with 10% FBS, 1% L-glutamine (Fisher Scientific Gibco L-Glutamine Solution Cytology 100 ml—25-030-081), 1% penicillin and streptomycin solution, 0.05 mM β-mercaptoethanol (Sigma, M6250), 0.11 g/L of sodium pyruvate (Sigma, P2256), and glucose 4.5 g/L (Sigma, G8644-100ML). THP-1 cells were kept in suspension with a maximum cell density of 800,000 cells per ml of medium. All the cell lines were kept in a humidified incubator at 5% CO2 pressure and 37*°*C (NuAire 200 L CO_2_ Incubator w/decon 120 V 60 Hz). To induce THP-1 cell differentiation, Phorbol 12-myristate 13-acetate (PMA) was purchased from Merck (P1585), and THP-e were incubated with 100 ng/ml PMA for 48 h. Human umbilical vein endothelial cells (HUVEC) were purchased from ATCC and cultured in a low serum medium (Biotechne, CCM027) using gelatin-coated cell culture plates and flasks. Gelatin derived from porcine skin was purchased from Merck (G1890-100G). To inflame HUVEC cells, lipopolysaccharide from *E. Coli* (LPS) was purchased from Merck (L4516-1MG). To stain cell nuclei, Hoechst 33258 solution was also purchased from Merck (94403). For membrane protein extraction, PIPES (P6757-25G), sucrose (S0389-100G), sodium chloride (S9888-500G), EDTA (E5134-50G), magnesium chloride (M8266-100G), digitonin (D141-100MG), and Triton X-100 (11332481001-50ML) were purchased from Sigma-Aldrich. Phosphate buffer saline (PBS 10X, pH 7.4, 70011044) was purchased from GIBCO. Protease inhibitor cocktail (Halt™ Protease Inhibitor Cocktail 100X) was purchased from Thermo Scientific. For NPs synthesis, ammonium sulphate was purchased from Sigma-Aldrich (A4418-100G), 1,2-Dipalmitoyl-sn-glycero-3-phosphocholine (DPPC) was purchased from Lipoid, 1,2-dioleoyl-sn-glycero-3-phosphocholine (DOPC, 850375P-200 mg) was purchased from Avanti Polar Lipids, cholesterol (C8667-500MG) was purchased from Sigma-Aldrich, and absolute ethanol (1009832511) was purchased from Merck. Float-A-Lyzers™ were purchased from Spectrum (Spectrum™ G235036); sterilizing 0.22 µm syringe filters were purchased from Merck (Z359904). To perform the Stewart assay, iron (III), chloride hexahydrate (F2877), ammonium thiocyanate (A0302), and chloroform (C2432-2.5L) were purchased from Sigma-Aldrich.

PDOs were extracted from CRC biopsies using the protocol summarized in the Supplementary Information and using the materials in Supplementary Table S1. Each PDO was obtained from a separate biopsy from a specific CRC patient.

### 2.2 Protocol for Membrane Protein Extraction

The protocol for membrane protein extraction was inspired by previous studies (Lehner et al., 2003). The buffers for NEXT are composed as follows:

• Washing buffer: 1X PBS.
• Extraction buffer 1 (EB1): 10 mM PIPES; 300 mM sucrose; 100 mM sodium chloride; 3 mM magnesium chloride; 5 mM EDTA; 0.015% w/v digitonin and 1:100 diluted protease inhibitor cocktail diluted in MilliQ water (pH = 6.8).
• Extraction buffer 2 (EB2): 10 mM PIPES, P6757 Sigma-Aldrich; 300 mM sucrose; 100 mM sodium chloride; 3 mM magnesium chloride; 5 mM EDTA; 0.1% or 0.5% v/v Triton X-100 and 1:100 diluted protease inhibitor cocktail diluted in MilliQ water (pH = 7.4).

We performed the membrane protein extraction on THP-1 cells, as schematized in Supplementary Figure S1, after removing them from the culture. In Step A, cells were rinsed twice with the wash buffers to remove the proteins present in the cell culture medium and centrifuged at 300 x g using an Eppendorf 5424R refrigerated centrifuge at 4°C. In Step B, cells were permeabilized by re-suspending them in EB1 at a ratio of 10^6^ cells per 100 µl of EB1, allowing the efflux of cytosolic and cytoskeletal proteins and incubated for 10 min. Cells were then centrifuged at 1,000 x g, and the supernatant was removed. In Step C, the pellets were re-suspended in EB2 to solubilize the cellular membranes and extract membrane proteins and were incubated for 30 min. The suspensions were then centrifuged at 5,000 × g to remove the cells’ nuclei and organelles. The supernatants containing the purified membrane proteins were finally recovered for further analysis. All the washing, centrifugation, and incubation passages were performed at 4°C. Membrane protein extracts were stored at −20°C.

Protein concentration in the extracts was determined using a BCA assay (Thermo Scientific™ Micro BCA™ Protein Assay Kit, 23235), according to the producer specifications. The proteins standard calibration curve was prepared using sequential dilutions of bovine serum albumin (BSA, Sigma—Aldrich, 100 g, 45ZV32) in the concentration range of 0–200 μg/ml. BSA dilutions were prepared in EB2 to account for possible interferences of the buffer components during the assay.

### 2.3 Nanoassemblr™ Formulation of Lipos and Leukos

Lipos and Leukos were formulated using the Nanoassemblr™ microfluidic equipped with its respective cartridges according to the producer’s instructions. The aqueous phase of the formulation was composed of a 250 mM ammonium sulphate buffer used to enable later DOXO remote loading. After dissolution, the buffer was filtered using a 0.22 µm filter and its pH was adjusted to pH 6.5 using a calibrated pH meter. For Leukos, membrane proteins were diluted into the buffer in concentration adjusted based on the FRR to achieve the desired lipids to protein ratio. On the other hand, the organic phase was a solution of DPPC, DOPC, and Cholesterol in a 4:3:3 molar ratio dissolved in absolute ethanol to a final concentration of 10 mg/ml. The two solutions were then sealed in glass vials and kept at 45°C using a heating block. During the formulation, the aqueous phase was loaded into a 3 ml syringe, while the organic phase was loaded in a 1 ml syringe. The temperature of the solutions during the formulation was kept by installing another thermal block within the Nanoassemblr™. Thus, the syringes were loaded into the system, and the Lipos or Leukos assembly was performed by setting the TFR, FRR (aqueous/organic), and lipids to protein ratio (w/w), as indicated by the DoE design. For the final formulations, the FRR was 4.88:1, the TFR was set to 1 ml/min, and the lipids to protein ratio was 20:1. The pre-waste of the system and the post-waste were always set as 50 and 150 µl, respectively. After synthesis, the NPs were dialyzed to remove the ethanol using a Float-A-Lyzer™ with a molecular weight cutoff of 300 kDa overnight at room temperature using PBS1X as an external phase. Finally, particles were filtered using a sterilizing 0.22 µm filter under a sterile laminar flow hood. Fluorescently labeled Lipos and Leukos were obtained by adding 0.05 mg Cy5.5 conjugated phosphatidylethianolamine (Cy5.5-PE) to the lipid phase before formulation (0.5% of the total lipid mass). To assess the particle fluorescence after formulation, Lipos-Cy5.5 or Leukos-Cy5.5 were diluted 1:1000 in MilliQ water, and then 100 µl of dispersion were added to a 96-well plate and fluorescence was measured using a Tecan Spar plate reader using an excitation wavelength of 620 nm (20 nm bandwidth) and an emission wavelength of 680 nm (30 nm bandwidth).

### 2.4 Stewart Assay for Lipids Quantification

The colorimetric Stewart assay was used to quantify the amount of lipids in the NPs formulations. In brief, the final NPs dispersion was diluted 1:50 in 2 ml of chloroform and thoroughly vortexed to endure complete lipids dissolution in the organic solvent. Then, 2 ml of Stewart aqueous reagent was added to the lipid solution, and the two phases were again thoroughly vortexed to allow for the reaction between the lipids and the reagent itself. The two phases were then separated by centrifugation for 10 min at 100 rpm. Then the reagent phase was removed, and the colored organic phase was spectrophotometrically analyzed at 485 nm wavelength. The concentration of lipids was calculated using a calibration curve to extrapolate the concentration from the net absorbance of the lipid solution. To create a calibration curve (Supplementary Figure S2), a freshly made chloroform solution of the lipids used for NP formulations in the same lipids’ ratio was diluted to 0.05, 0.04, 0.03, 0.02, 0.01, and 0.005 mg/ml of total lipids and pure chloroform as a blank. The calibration curve and its relative equation are presented in the graph below.

### 2.5 DLS Characterization of Lipos and Leukos

Lipos and Leukos hydrodynamic diameter, PDI, and zeta potential were measured using a Zetasizer DLS (Malvern). Particles’ solutions (1.2 mg/ml of lipids) were diluted 1:100 in fresh MilliQ water and measured using folded capillary cuvettes (DTS1070, Malvern) five sequential times for the size, followed by three measurements of zeta potential, each interspaced by one-minute equilibration time.

### 2.6 Particle Tracking Analysis of Lipos and Leukos

The number of particles per milliliter of the formulation was estimated using the Nanosight™ platform equipped with a 488 nm laser. Lipos or Leukos were diluted 1:1000 in fresh MilliQ water prior to the analysis and then loaded into a 1 ml syringe. For the analysis, the particles were injected at 1000-speed for 10 s, followed by 120 s of 100-injection speed prior to measurement. Each sample was measured while injected at a 100-injection speed in five sequential measurements of 60 s each, keeping the camera level at 12. For data processing, the detection threshold was set at 7 for all measurements.

### 2.7 Flow Cytometry Analysis and MACSPlex Exosome Assay

Human monocytic leukemia cell line THP-1 was analyzed by cell surface phenotyping using the BD FACSymphony (Becton Dickinson, San Diego, CA, United States). In detail, THP-1 cells were counted, and up to 0.5 × 10^6^ cells were stained at 4°C with a selected panel of immune cell markers (mouse anti-human monoclonal antibodies CD45, CD19, CD3, CD4, CD8, CD56, CD11c, CD14, HLA-DR, HLA-ABC, CD86, and CD1c: all from BD Biosciences). A marker of cell viability (BD Horizon™ Fixable Viability Stain 780) was also added.

Leukos derived from THP-1 cells were characterized by cytofluorimetric analysis using the MACSPlex exosome kit (Miltenyi Biotec, Bergisch Gladbach, Germany) following the manufacturer’s instruction. In brief, three different Lipo and Leuko preparations (1 × 10^10^ particles) were added to the MACSPlex buffer solution. Samples were then incubated for 2 h with a cocktail of APC-conjugated detection antibodies against tetraspanins (CD9, CD63, and CD81) and phycoerythrin (PE)- and fluorescein isothiocyanate (FITC)-labeled capture beads, coated with antibodies against 37 different exosomal surface epitopes plus two isotype controls (REA and IgG1). Beads and detection antibodies were incubated without particles in the MACSPlex buffer and used as a negative control. After washing steps, samples were analyzed by flow cytometry using the BD FACSCanto II (Becton Dickinson, San Diego, CA, United States), capable of detecting the necessary fluorescence signals. To eliminate a non-specific signal from the median fluorescence intensity (MFI) of each marker was subtracted the MFI of the negative control as well as of the isotype controls used in the same experiment. Leukos specific signal was then obtained by subtracting the corresponding Lipos marker signal in each preparation. All the data were analyzed by using FlowJo software version 10.8.1 (Becton Dickinson, San Josè, CA, United States).

### 2.8 Doxorubicin Loading Into Lipos and Leukos

DOXO loading was performed using a remote loading approach. Specifically, DOXO hydrochloride was dissolved in fresh MilliQ water to a final concentration of 1 mg/ml. Then, the DOXO solution was added to the NP suspension according to the desired Lipids to DOXO weight ratio, according to the following formula:

$$\text{Vol of DOXO to ad }\left(\text{mL}\right)=\;\frac{\left(\text{Lipids conc }\left(\frac{\text{mg}}{\text{mL}}\right)\text{.Final vol }\left(\text{mL}\right)\right)}{\left(\text{Lipids to DOXO ratio}\text{.DOXO conc }\left(\frac{\text{mg}}{\text{mL}}\right)\right)+\text{Final vol }\left(\text{mL}\right)}$$ (1)

The DOXO and NP mixture was then incubated in a thermal block at 45°C for 2 h under 300 rpm stirring. Then, the NPs were dialyzed using a Float-A-Lyzer™ with a molecular weight cutoff of 300 kDa overnight at 4°C using PBS1X as an external phase to remove the DOXO that was not loaded within the particles while avoiding drug leakage.

### 2.9 Assessment of Doxorubicin Loading

DOXO quantification was performed using the intrinsic drug fluorescence. A Tecan Spark plate reader was used to measure the drug fluorescence by excitation at 485 nm and emission at 590 nm. A calibration line of DOXO in MilliQ water was created by using DOXO solutions in the range of 0–2 μg/ml (Supplementary Figure S3). To calculate DOXO encapsulation efficiency within the particles, we used a fluorescence-dequenching assay, in which Triton X-100 was used as a detergent to disassemble the nanovesicles and release the encapsulated DOXO sulphate crystals, which would then dissolve in water and restore the DOXO fluorescence that would otherwise be quenched in its crystalline form. NPs were diluted 1:2 in a 0.2% (v/v) solution of Triton X-100 in MilliQ water and stirred for 10 min. Then, 100 µl solution was added in triplicate in a 96-well plate, and the reading was performed according to the previously reported plate reader settings. The encapsulation efficiency was calculated as follows:

$$\text{Encapsulation Efficiency }\left(\text{\%}\right)=\;\frac{\text{DOXO loaded in the NPs }\left(\text{mg}\right)}{\text{Initial amount of DOXO incubated }\left(\text{mg}\right)}.100$$ (2)

### 2.10 Assessment of Doxorubicin Release

DOXO release was performed by diluting Lipos or Leukos 1:50 in 2 ml of 1X PBS supplemented with 10% FBS (v/v) to simulate the cell culture medium. The release medium pH was either kept at pH 7.4 or adjusted to pH 6 to simulate the lysosomal compartment. The NP dispersion was then incubated in a thermal block at 37°C under 300 rpm stirring. At different time points, 50 µl of the suspension were withdrawn and diluted either in water or in 0.2% Triton X-100 to measure the released DOXO and the total DOXO within the aliquot, respectively, using the same fluorimetric assay discussed in the previous section. The released DOXO percentage was calculated by dividing the DOXO measured in water over the total DOXO within the aliquot, multiplied by 100.

### 2.11 *In Vitro* Cytotoxicity Assays

To perform a cytotoxicity assay of DOXO and NPs, HCT-116 were seeded in a 96-well cell culture plate at a density of 10,000 cells per well and left to adhere overnight in a complete culture medium. The day after, the medium was withdrawn from the cells and replaced with either free DOXO, Lipos, Leukos, Lipos-DOXO, or Leukos-DOXO in a dose range from 10 to 0.001 µM. The amount of DOXO loaded particles added was calculated based on the amount of DOXO they contained to make the treatment analogous to the free drug. Conversely, empty Lipos and Leukos were used in the same NP number as their respective DOXO loaded formulations. Then cells were incubated for 72 h at 37°C with 5% CO_2_. Afterwards, 20 µl of Resazurin solution (400 µM) were added to each well and incubated for 2 h. Finally, the fluorescence was measured using a Tecan Spark plate reader with excitation at 550 nm and emission at 590 nm. The percentage of cell viability was calculated as the ratio between the fluorescence over the fluorescence of the untreated cells, multiplied by 100. After normalizing the viability data, the half-maximal inhibitory concentration (IC_50_) values for each treatment were calculated using GraphPad Prism to fit a sigmoidal curve of the percentage of viable cells versus the decimal logarithm of the treatment concentration expressed as µM.

For the assessment of NPs cytotoxic effect on PDOs. PDOs were seeded in Geltrex™ at a density of 50,000 cells per 20 µl of gel and kept in culture for 72 h before receiving treatment. Then, PDOs were treated with Lipos, Leukos, Lipos-DOXO, or Leukos-DOXO as indicated for the HCT-116 organoids. Cell viability was assessed using the Cell Titer-Glo^®^ 3D Cell Viability Assay according to the manufacturer’s indications. The IC_50_ values were calculated as indicated for HCT-116 cells.

### 2.12 *In Vitro* Nanoparticles Uptake Assay

To assess the NPs uptake, HCT-116 were seeded in culture in the same conditions discussed in the previous section. Human umbilical veins endothelial cells (HUVEC) were seeded on gelatin-coated 96 well plates at 10,000 cells per well density. To activate endothelial cells, they were incubated with a 100 ng/ml solution of LPS in a complete medium for 3 h. Human THP-1 cells were instead seeded in 24-well plates as 50,000 cells per well in the presence of PMA 100 mg/ml and left to differentiate into macrophage/like cells for 48 h. PDOs were seeded in Geltrex™ at a density of 50,000 cells per 20 µl of gel and kept in culture for 72 h before treatment. The cell culture medium was then replaced with fluorescent Lipos Cy5.5 or Leukos Cy5.5 dispersed in a complete cell culture medium at a final concentration of 1 × 10^11^ particles/ml. HCT-116 cells were then incubated for 6, 12, and 24 h before imaging, while HUVEC and THP-1 cells were incubated for 3 h before imaging. At the time of imaging, the cells culture medium was removed, and cells were washed with 1X PBS and stained for their nuclei by incubating them with a Hoechst solution of 20 µM for 5 min at 37°C. Cells were then imaged using a Zeiss AxioObserver fluorescence microscope equipped with a TL lamp for bright field imaging with a 385 nm laser for Hoechst visualization and a 630 nm laser for Cy5.5 detection. To perform the quantification of NP uptake, an *ad hoc* macro was created in FIJI to determine the NPs fluorescence and the number of cells per image, following the workflow schematized in Supplementary Figure S4. Then, the NP fluorescence was normalized by the number of cells in its respective image, as presented in Supplementary Figure S4.

### 2.13 Statistical Analysis

We performed every experiment in triplicate, and statistically significant differences were assessed using two-way ANOVA analysis for matching values.

## 3 Results and Discussion

### 3.1 Leuko Optimization by Design of Experiment and Characterization

To produce Leukos, the Nanoassemblr™ platform was selected. This instrument is based on a microfluidic chip in which two liquid, miscible phases are blended very quickly and efficiently by converging into a “herringbone” mixing device. The mixing of the two phases thus occurs within milliseconds. The principle upon which the Nanoassemblr™ allows the formulation of liposomes relies on the solubilization of lipids or hydrophobic polymers within the organic phase. When this solution mixes with the aqueous phase within the system, the overall polarity of the organic phase suddenly decreases due to the introduction of water. In turn, this increased polarity increases the free energy of the system and induces the self-assembly of the hydrophobic lipids into liposomes. This self-assembly combined with the shear stress caused by the microfluidics components breaks down the newly formed vesicles into nanovesicles with a narrow size distribution (Molinaro et al., 2018).

Considering this approach for the formulations of Leukos, their hydrophobic components are specifically phospholipids and cholesterol, specifically DPPC, DOPC, and cholesterol. Among the many different lipid mixtures employed in the formulation of liposomes, a 4:3:3 molar ratio of DPPC: DOPC: Cholesterol, as indicated in the literature, was used (Molinaro et al., 2018). Thus, this mixture of lipids was dissolved in absolute ethanol at a total concentration of 10 mg/ml. Ethanol was selected as a solvent since it is compatible with the Nanoassemblr™ cartridge material, is easily miscible with water and removable *via* dialysis, and is relatively biocompatible in the case some residues are still present within the final formulations.

On the other hand, the aqueous phase of Leukos is composed of a dispersion of membrane proteins derived from the previously optimized cell extracts in a 250 mM ammonium sulphate buffer. This buffer was selected from previous studies to enable the loading of DOXO using the remote loading approach (Barenholz, 2012). During the process of self-assembly occurring in the microfluidics system, lipid nanovesicles and the integration of membrane proteins in their phospholipid bilayer in a single step, ultimately forming Leukos.

Therefore, the main controllable experimental parameters to be optimized in this process are as follows:

• Total flow rate through the system (TFR), which is the speed of the overall flow of both phases within the Nanoassemblr™, expressed in ml/min.
• Flow rate ratio (FRR) between the aqueous and the organic phase, specifically the ratio between the speeds of the organic phase over the flow rate of the aqueous phase.
• The weight ratio between membrane proteins and lipids (Lip/Prot ratio). The mass of lipids was kept constant for simplicity, while the number of proteins in the aqueous phase was modulated to a specific ratio and was further adjusted by considering the FRR. For the formulation of Leukos, THP-1 cells human membrane protein extracts were employed.

**FIGURE 1:**
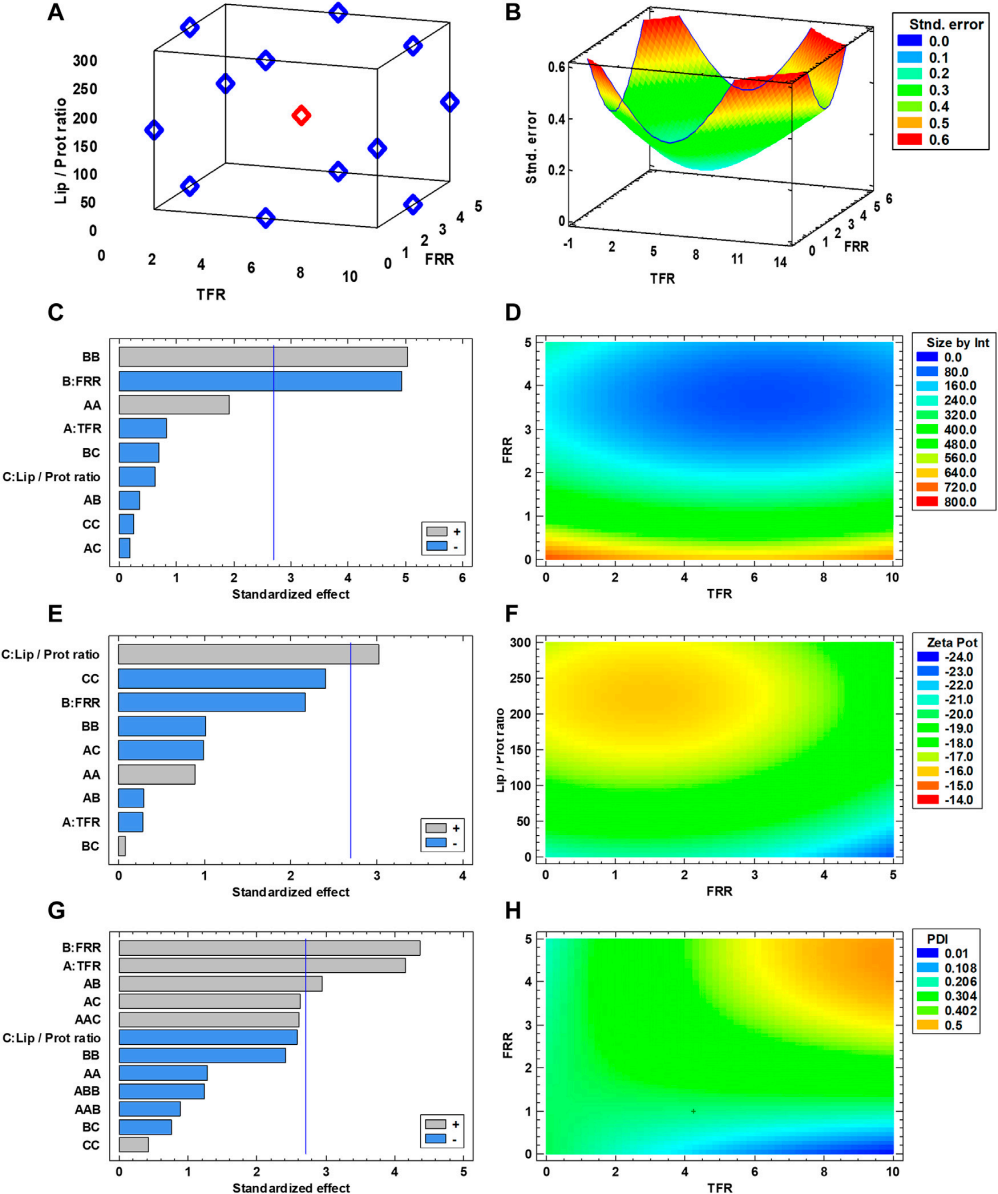
**(A)** Schematic representation of the DoE used for Leuko optimization. **(B)** Analysis of variance used for the DoE. **(C,E,G)** Standardized Pareto Chart for statistical significance of the experimental parameters, red lines represent *p* = 0.01. **(D,F,H)** Response surfaces fitted for each analyzed response (*n* = 3 for all the performed experiments).

To create the DoE for screening and optimization, the selected range of values for the experimental parameters was as follows: TFR between 1 and 10 ml/min, FRR from 1:1 to 5:1 (aqueous/organic phase), and Lip/Prot ratio from 300:1 to 20:1. Using the Stat Graphics Centurion XIX software, a three-level, randomized, Box-Behnken design with three complete replicates and a center point was generated (Commerce, 2013). The entire set of combinations for a single replicate is listed in Supplementary Table S2, whereas the visual representation of the design space is presented in Figure 1A. The prediction variance analysis graph (Figure 1B) demonstrated a completely symmetrical trend in variance with an increasing pattern from the design space center, making the design rotatable. This indicates how the accuracy of the predictions offered by DoE tends to decrease from the center of the experimental space. Furthermore, the symmetry of the variance represents how the design is, *per se*, unbiased in any direction, giving similarly accurate predictions across all the dimensions of the experimental space. After performing all the experimental runs, all Leuko formulations were dialyzed against PBS to remove ethanol and were characterized using DLS to measure their average size by intensity, their polydispersity index (PDI) and their zeta potential. Zeta potential was used as a proxy for membrane protein integration since the previous studies on similar formulations evidenced how the integration of membrane proteins onto Leukos decreases the particle’s zeta potential compared to liposomes with the same composition (Molinaro et al., 2016; Martinez et al., 2018; Molinaro et al., 2020). Using the software, the experimental results were analyzed to understand the influence of experimental parameters or their combinations on the different responses taken into consideration. For the size by intensity, the only relevant factor appeared to be FRR (Figure 1C), whose increase, decreased the particle size. For zeta potential, as expected, the most relevant variable was the lipids/proteins (Lip/Prot) ratio (Figure 1E); specifically, increasing the ratio also increased the zeta potential and vice versa, therefore making it more negative with the increase in proteins. Finally, PDI was influenced by both TFR and FRR, and interestingly, their combination also (Figure 1G) increased with them. The experimental data were then interpolated to create response surfaces for the results, which allowed seeing the overall trend in each one depending on the selected variables (Figures 1D, F, and H). The creation of response surfaces allows the extrapolation of optimal values of experimental factors for a specific desired outcome.

In the case of Leukos formulation, the desired features for these particles are as follows:

• A diameter in the nano range (between 20 and 200 nm specifically) that allows for their long circulation in the blood after IV injection (Yao et al., 2020). For this formulation, the desired size of 150 nm was selected as the target.
• A low PDI (around or below 0.2) that makes the formulation homogeneous in size and thus with reproducible features and behavior. Thus, the selected PDI response was the lowest value within the tested range (Danaei et al., 2018).
• A low zeta potential. This could appear counterintuitive since most of the literature shows how neutral or slightly negatively charged particles are normally the ones with the longest circulation time (Zinger et al., 2021; Molinaro et al., 2018, Design and Development of Biomimetic Nanovesicles Using a Microfluidic Approach. 2018). However, as previously stated, the surface charge of our particles was used as an indication of the presence of membrane proteins on their surfaces. Thus, a minimized zeta potential would correspond to a high protein loading efficiency.

After providing all these formulations to the software, the calculated optimized values were a TFR = 1 ml/min, a FRR = 4.88:1, and a Lip/Prot ratio of 1:20. The software also predicted the expected values of size, PDI, and zeta potential at 150 nm, 0.31, and −25 mV, respectively. The overall optimization yielded an overall desirability of 85% of the selected responses (Supplementary Figure S5). To confirm the validity of these predictions, the optimized parameters were used to formulate the actual Leukos. As a negative control, Lipos with the same lipid composition but without membrane proteins in their formulations were produced. Each time, Lipos and Leukos were prepared starting from the same lipid batches and buffers. This control not only allows constant maintenance of all the features of the formulation but also enables evidence of possible discrepancies in NPs behavior and features that can be ascribed to the presence of the membrane proteins themselves. After performing these experimental runs, Lipos and Leukos were analyzed using DLS.

**FIGURE 2:**
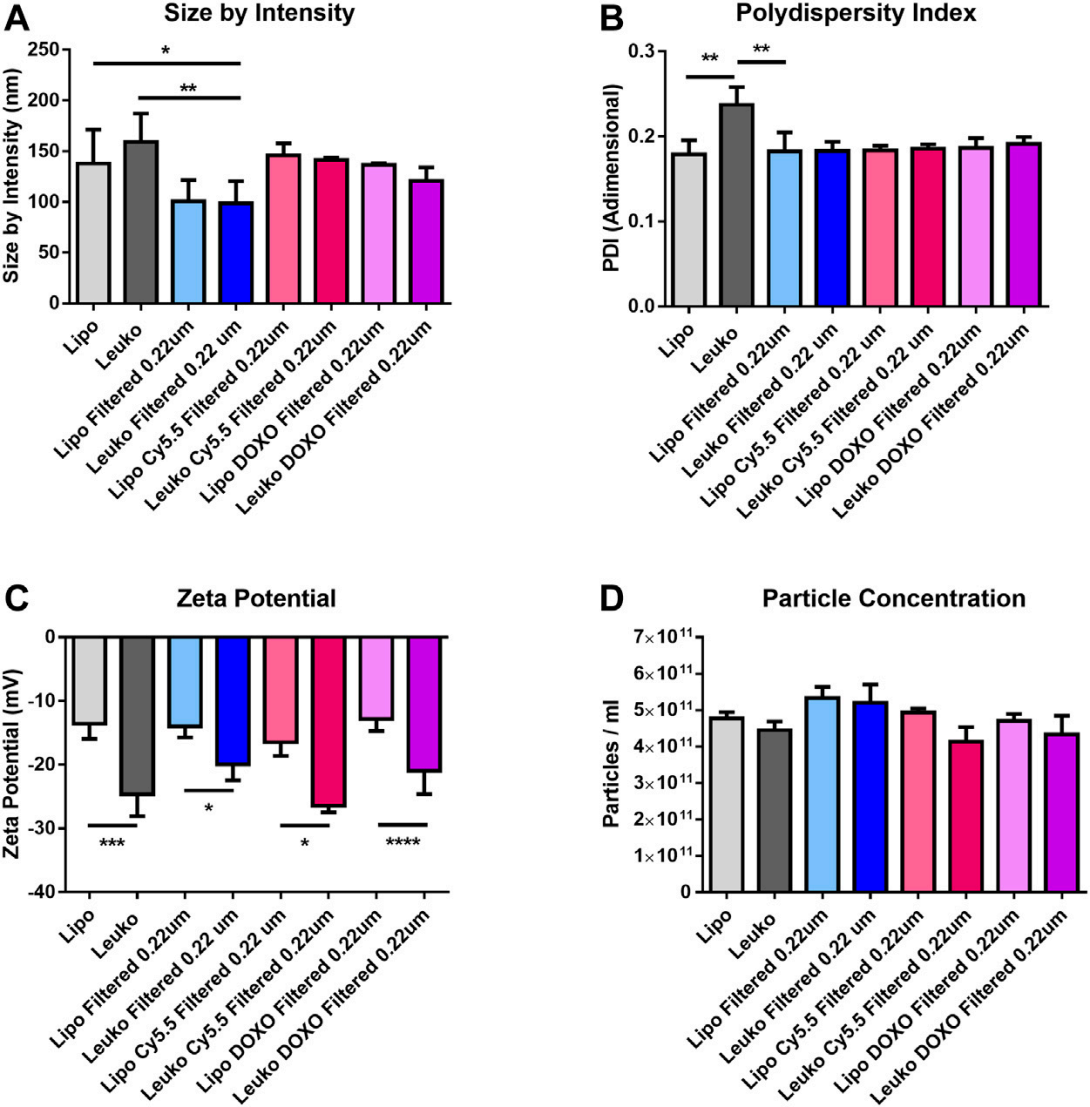
Size by intensity **(A)**, polydispersity Index (PDI) **(B)**, and zeta potential **(C)** measured by DLS for the different Lipo and Leuko formulations. **(D)** Particles concentrations estimated by particle tracking analysis using the Nanosight™ platform. (*p* ˂ 0.05; ∗∗*p* ˂ 0.01; ***p ˂ 0.001; ∗∗∗∗*p* ˂ 0.0001, and *n* = 3 for all the performed experiments).

**FIGURE 3:**
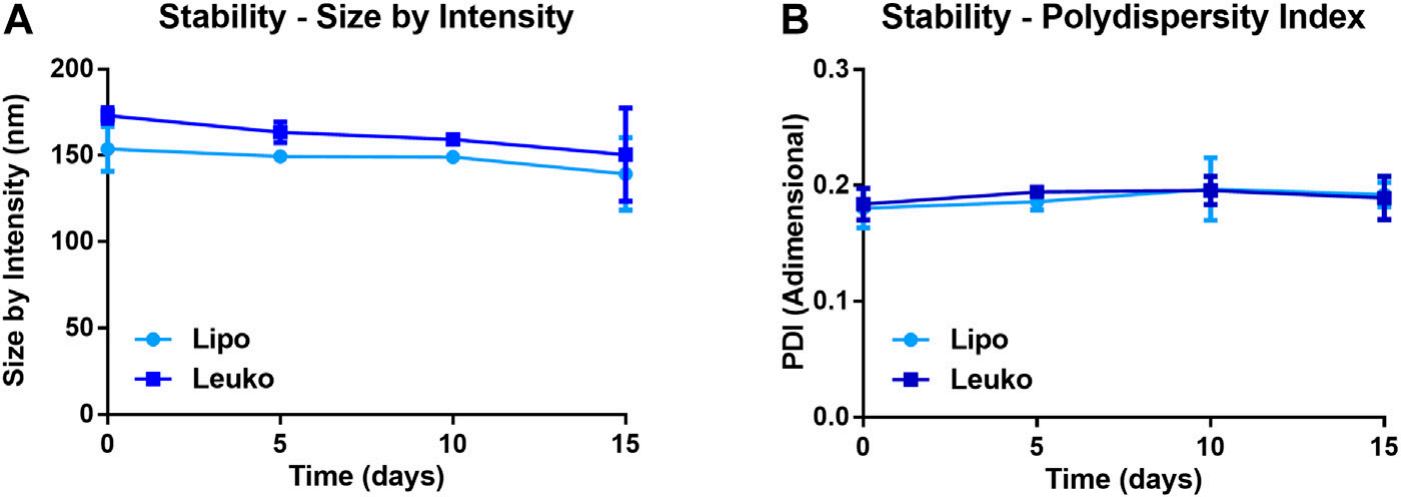
Size by intensity **(A)** and PDI **(B)** of Lipos and Leukos measured over 15 days of storage in PBS solution at 4°C. (*n* = 3 for all the performed experiments).

Figure 2A shows that the average size by intensity for both Lipos and Leukos was around 137 ± 34 nm and 159 ± 28 nm, respectively. Conversely, the PDI was 0.179 ± 0.017 for Lipos, while it was slightly higher for Leukos (0.237 ± 0.021 Figure 2B). Finally, the zeta potential for Lipo was set around −13.6 ± 2.4 mV, while for Leuko, it was significantly lower (−24.7 ± 3.4 mV, Figure 2C). Taken together, these data confirm the reliability of our predictions as calculated by the DoE-derived mathematical model. The number of particles per ml of suspension was also measured using nanoparticle tracking analysis (NTA) *via* the Nanosight™ equipment. As presented in Figure 2D, Lipos and Leukos preparations had 4.8 ± 0.17 × 10^11^ particles/mL and 4.45 ± 0.24 × 10^11^ particles/ml, respectively. The PDI value of Leukos was still considered to be above the acceptable threshold of 0.200. To reduce it, particles after dialysis were filtered using a PVDA filter with a 0.22 µm cutoff. This procedure has the dual convenient effect of removing the big particle aggregates from the suspensions, ultimately reducing the PDI, and sterilizing the particles when performed in sterile conditions. Interestingly, the filtration managed to decrease the size of both Lipos and Leukos down to 100 ± 21 nm and 99 ± 22 nm. The PDI was lowered to 0.186 ± 0.011 for Leukos, and, at the same time, the difference between the zeta potential of Lipos (−14.0 ± 2.4 mV) and Leukos (−22.3 ± 2.5 mV) and their particles number (5.3 ± 0.3 × 10^11^ particles/ml and 5.2 ± 0.5 × 10^11^ particles/ml) were retained, despite a small reduction in the absolute Leukos surface charge. Finally, fluorescent Lipos and Leukos (Lipo Cy5.5 and Leuko Cy5.5, respectively) were formulated by adding to the lipidic phase a fraction of Cy5.5 conjugated phosphatidylethanolamine (Cy5.5-PE), corresponding to 0.5% of the total lipid mass used for the formulation. These formulations were also characterized using DLS and NTA, confirming that their size, PDI, zeta potential, and particle number were consistent with the other formulations. Finally, the Cy5.5 fluorescence of labeled Lipos and Leukos was measured using a plate reader. For both formulations, the fluorescence was very consistent. This feature is important to later quantify these particles’ uptake in later *in vitro* uptake on cell cultures (Supplementary Figure S6). Lipos and Leukos stability under 4°C storage conditions were also assessed with DLS over 15 days, demonstrating that the NPs size and PDI did not change significantly over time (Figures 3A and B, respectively).

**FIGURE 4:**
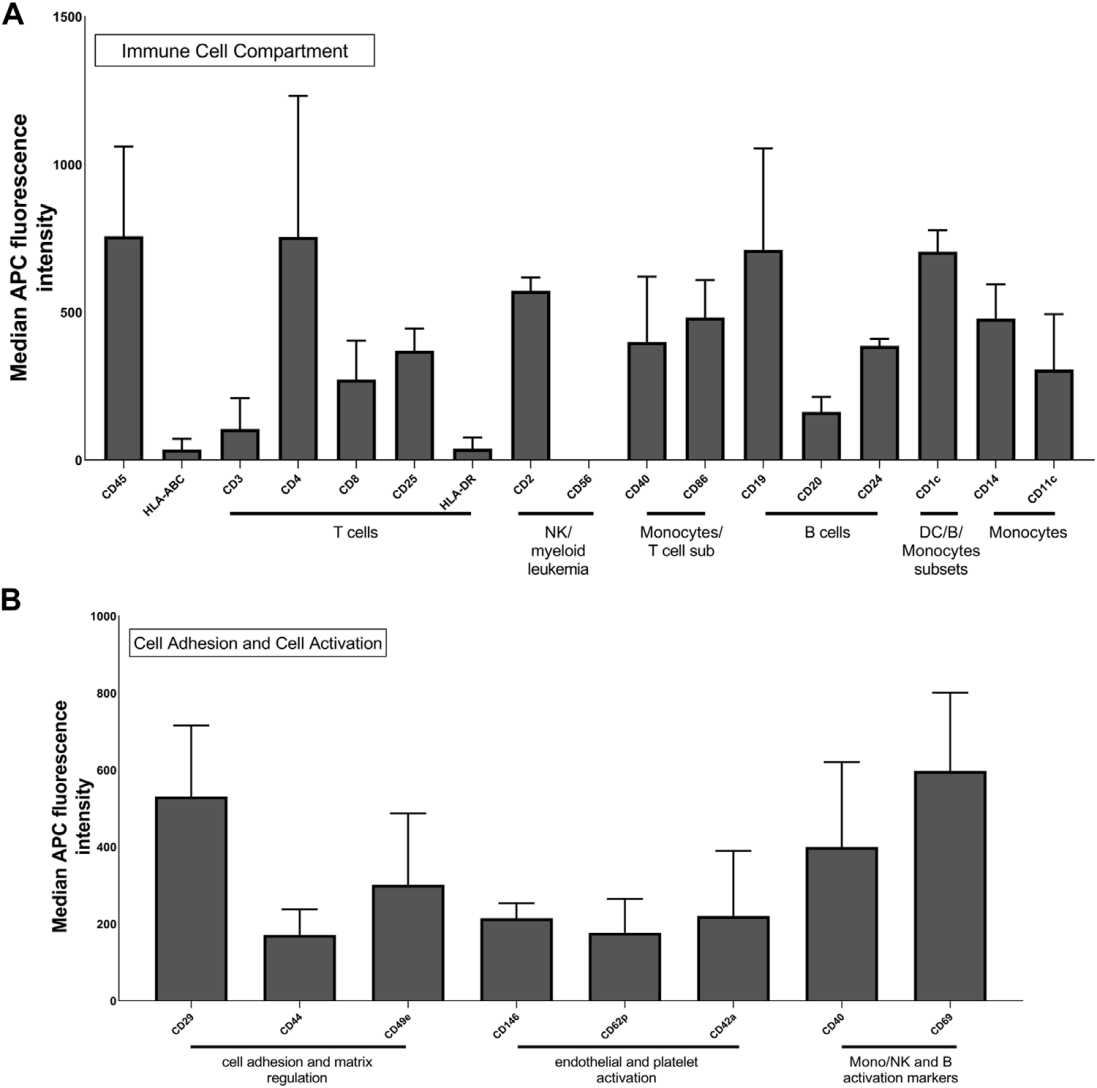
Multiplex flow cytometry analysis of Leuko markers for detection of immune cells **(A)** and molecules involved in cell adhesion and activation **(B)**. The x-axis shows the protein marker profile, whereas the y-axis represents the normalized APC-MFI. The median APC signal intensity of each specific Leuko surface marker was normalized to the median of each Lipo surface marker. MFI, median fluorescence intensity.

To assess the presence of membrane proteins on the surface of Leukos, these particles were analyzed for different immune cell surface markers by flow cytometry. Cytofluorimetric analysis of THP-1 Leukos originating cells was also conducted to evaluate their phenotype. As presented in Supplementary Figure S7, THP-1 cells presented a mixed phenotype in terms of immune markers, resulting positive for general leukocyte markers (CD45) (Al Barashdi et al., 2021), markers for T cells (including CD3, CD8, and HLA-DR) (Golubovskaya and Wu, 2016), markers for NK cells (CD56) (Del Zotto et al., 2017), for monocytes (CD86) (Kapellos et al., 2019), and for B cells (CD19) (Sanz et al., 2019). These data corroborate the derivation of THP-1 cells from an acute monocytic leukemia clone and their aberrant expression of a mixture of monocytic and leucocytic surface markers (Hsu and Hsu, 1989). As shown in Figure 4A, Leukos presented on their surface all these markers, and remarkably, almost all the markers analyzed on THP-1 cells were strongly retained onto leukosomes (Supplementary Table S3).

Despite the difficulties in directly correlating the cytofluorimetric results from cells and from Leukos, we can assert that the analyzed markers were successfully translated from cells to the Leukos surface. It is also important to note, as presented in Figure 4B, that Leukos also present on their surface, proteins involved in vessels adhesion (CD29 and CD44) (Ponta et al., 2003; Brakebusch and Fä ssler, 2005), which are paramount for their efficient adhesion to the endothelial cells lining the inflamed blood vessels and molecules related to monocytes activation (CD40 and CD69) (Elgueta et al., 2009; Cibrián and Sánchez-Madrid, 2017; Guezguez, 2006).

Furthermore, since all the antibodies are normally used to identify markers on the surface of cells and EVs, they are specific for the extracellular domains of the proteins and thus confirm both the presence and the correct orientation of specific molecules. Previous studies using similar flow cytometry and computational approaches demonstrated how most proteins onto Leukos are correctly oriented due to a mixed effect of steric hindrance and lipids membrane curvature (Molinaro et al. 2018, E. M. Molinaro R, Design and Development of Biomimetic Nanovesicles Using a Microfluidic Approach. 2018).

### 3.2 Doxorubicin Loading Into Lipos and Leukos Using a Remote Loading Strategy

DOXO is an antitumor drug used against many different neoplasms. This molecule can exert its cytotoxic effect *via* multiple molecular mechanisms, including DNA intercalation, induction of oxidative stress, and activation of several signaling pathways that, in turn, lead to cell death (Yaqub, 2013). Furthermore, this drug has a low molecular weight (543,52 g/mol), and thus DOXO can easily permeate the cell membrane to reach the intracellular molecular target *via* diffusion. Moreover, it is very soluble in water, a feature which allows the administration of high doses *via* intravenous injection.

However, many tumors are characterized by the development of multidrug resistance. This phenotype can be enabled by several mechanisms, including the overexpression or mutation or drugs intracellular target, the use of alternative signaling pathways to evade or compensate for the drug action, or even by the expression of extrusion pumps onto the plasma membrane that quickly remove the drug from the cytosol, significantly reducing their cytotoxicity (Rueff and Rodrigues, 2016). Since DOXO is a traditional chemotherapeutic with multiple and pleiotropic effects on cell biology, the first two mechanisms of resistance mentioned above are somewhat overcome by this molecule. However, DOXO is still a substrate for extrusion pumps. When DOXO is loaded within NPs, its uptake follows the same mechanism of the particles, which are normally internalized by endocytosis or phagocytosis, depending on their size, shape, surface proprieties, and on the target cell in the study (Sousa de Almeida et al., 2021). Remarkably, DOXO has been previously encapsulated with success into liposomes in already clinically approved nanoformulations, including the first approved NPs for the treatment of tumors (DOXIL™) and their PEGylated version (CAELYX™) using a remote loading approach.

This technique allows the fast, reliable and efficient loading of DOXO and other small molecules drugs by creating a pH gradient between the external environment (pH = 7.4) and the internal aqueous core of nanovesicles (pH = 6.5). DOXO is a small molecule with low molecular weight, allowing DOXO to permeate the vesicle’s phospholipid bilayer and reach the hydrophobic core. However, when DOXO molecules meet the acidic pH within liposomes, they become positively charged, losing the ability to permeate the phospholipid bilayer due to their increased polarity. This mechanism is normally defined as “ionic entrapment.” Furthermore, the ionized DOXO molecules in the presence of sulphate ions spontaneously precipitate into DOXO sulphate nanocrystals within the nanovesicles. The combination of these equilibria has the net effect of “pulling” DOXO molecules within the liposomes in a high amount. Furthermore, the formation of DOXO sulphate crystals allows the packing of a high amount of drug within a very small volume, ultimately leading to high encapsulation efficiency (Fritze et al., 2006).

DOXO loading thus was performed by testing different ratios between the amount of Lipos or Leukos, expressed as lipid concentration calculated using the Stewart colorimetric assay, and the amount of DOXO used. The incubation was performed by simply mixing the empty NPs suspension and DOXO solution (1 mg/ml) and keeping it under stirring for 2 h at 37°C, according to most protocols used in the literature (Molinaro et al., 2020). After incubation, the DOXO that was not encapsulated was removed by dialysis overnight at 4°C, using a Float-A-Lyzer™ system with a 300 kDa molecular weight cutoff. The low temperature of dialysis was used to avoid drug leakage from the particles during dialysis, keeping the lipids from composing Lipos and Leukos in their “solid” state. After DOXO loading, the particles were again characterized *via* DLS and Nanosight™, confirming no significant changes in their features compared with empty Lipos and Leukos.

The amount of DOXO loaded within the particles was then calculated using a fluorimetric assay. DOXO is characterized by a high intrinsic fluorescence, which is linearly proportional to its concentration within a limited range.

However, DOXO loaded within Lipos and Leukos is present mostly in crystal form, which quenches its fluorescence significantly compared to the free drug (Molinaro et al., 2020). Thus, detergents are normally used to disrupt the particles membranes. This causes the dissolution of the DOXO sulphate crystals and de-quenches DOXO fluorescence, making it measurable. This approach thus is normally termed as “fluorescence dequenching.” To disrupt our Lipos and Leukos, 0.2% v/v Triton X-100 mixed in a 1:1 volume ratio to a final concentration of 0.1% Triton X-100 was used. After a short duration of stirring, DOXO fluorescence was then measured, and the encapsulated DOXO was calculated using calibration lines for DOXO dissolved in water or 0.1% Triton X-100 and divided by the initial amount of incubated DOXO to calculate the encapsulation efficiency, expressed as mg of encapsulated DOXO/initial DOXO•100.

**FIGURE 5:**
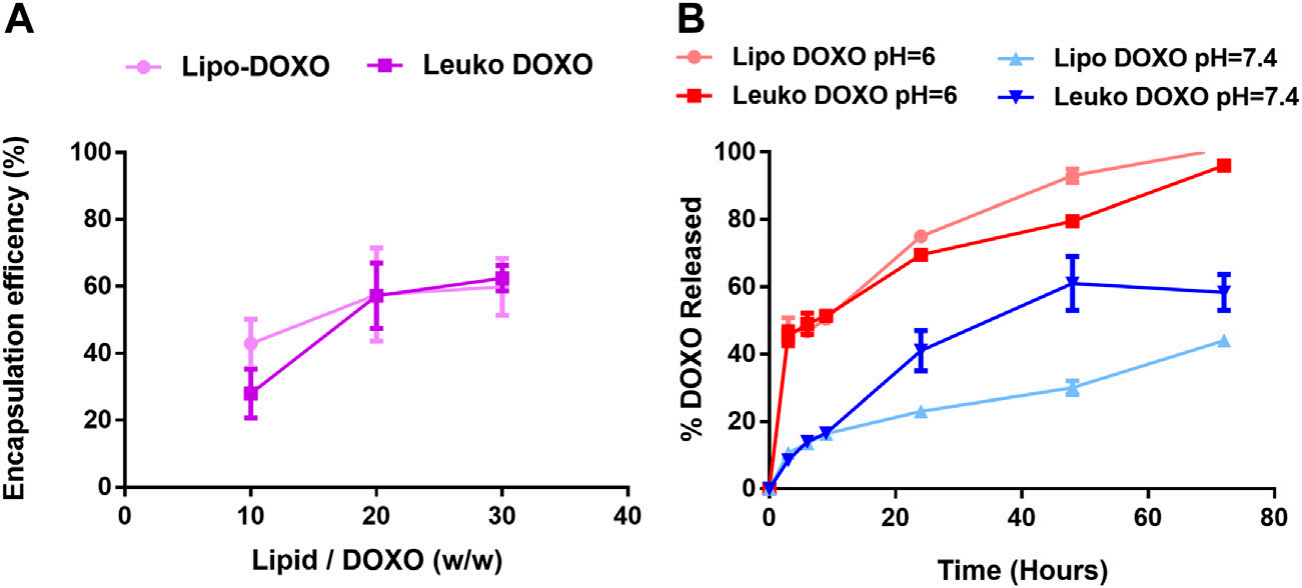
**(A)** Encapsulation efficiency of DOXO profile for Lipos and Leukos at different lipids to DOXO ratios. **(B)** DOXO release profile from Lipo-DOXO and Leuko-DOXO at pH = 7.4 and pH = 6 (*n* = 3 for all the performed experiments).

As presented in Figure 5A, the tested lipids to DOXO ratio for drug loading were 10:1, 20:1, and 30:1 (w/w). The encapsulation efficiency tended to increase with the increase of the ratio. This may appear counterintuitive since decreasing the relative amount of DOXO results in more of it being loaded. However, the available space for DOXO encapsulation within the nanovesicles is a very small fraction of the overall NPs suspension. Thus, incubating a high amount of drug would lead to reaching a maximum of encapsulated DOXO amount, with a high fraction of the drug that is in excess and not successfully loaded within the NPs. Thus, by decreasing the total amount of DOXO, it is possible to reduce this excess without compromising the amount of loaded drug, ultimately increasing the encapsulation efficiency. The lipids to DOXO ratio that was selected for further investigation was 20:1 since it yields the highest encapsulation efficiency without reducing the DOXO amount too much (55% ± 11% for Lipos and 56% ± 7% for Leukos).

Of note, CRC chemotherapy protocols do not include doxorubicin (Gustavsson et al., 2015). DOXO was selected as a drug that has been efficiently encapsulated in well-established and clinically approved liposomal formulations (Doxil™ and Caelyx™) using the remote loading approach to achieve high encapsulation efficiency and loading. This allowed a close comparison between Leukos and the mentioned well-established and studied nanovectors. Indeed, compared to similar leukosomes formulations (Molinaro et al., 2020), the loading efficiency appears to be lower. However, these formulations were formulated using the traditional thin layer hydration approach and had a lipid composition with a higher fraction of saturated lipids. This makes Lipos and Leukos phospholipid bilayers more rigid, preventing DOXO leakage during dialysis and promoting higher drug retention.

We believe these results would pave the way not only for the encapsulations of other drugs used for CRC chemotherapy but also for other pathological contexts, with particular attention to anti-inflammatory drugs that are especially good candidates as therapeutic payloads for leukosomes and have already been loaded into nanovesicles using a similar pH driven loading process (Al-Amin et al., 2020).

### 3.3 Doxorubicin Release Profile From Lipos and Leukos

After optimizing DOXO loading, the next step was to assess its release kinetics. To assess this, DOXO loaded Lipos and Leukos were diluted 1:50 in PBS 1X supplemented with 10% v/v of FBS. The presence of FBS was necessary to better replicate the composition of the cell culture medium used in further studies. Conversely, PBS 1X was used to replace the cell culture basal medium since it contains a high amount of phenol red, a pH indicator, which is intrinsically fluorescent and could cover the DOXO signal.

The release was tested for Lipos and Leukos at pH 7.4 to simulate the physiologic pH of the cell culture media and biological fluids and at pH 6.0 to simulate the conditions of the intracellular lysosomes after NPs uptake. As presented in Figure 5B, DOXO release for Lipos and Leukos at pH 6.0 was quite fast, and the complete release was achieved within 72 h. Conversely, the release was significantly slower for both NPs formulations at physiological pH. This is especially useful since after the particles are internalized by cells *via* endocytosis, they are normally trafficked to the lysosomal compartment, in which the pH reaches values down to 5.5. In these conditions, the DOXO crystals dissolve, and the drug is released within the cell’s cytosol, where it can exert its cytotoxic effect. Conversely, at the physiologic pH value of 7.4 found in the blood circulation, DOXO is retained within the particles, avoiding the unwanted leakage of the drug during NPs circulation that could lead to systemic off-target adverse effects. However, it is notable that the release of DOXO from Leukos is significantly faster than bare Lipos. This could be due to the presence of membrane proteins on the surface of Leuko, which could partially disrupt the phospholipids and cholesterol bilayer, making the drug leakage slightly faster.

Nevertheless, it is important to note that most DOXO is retained by both Lipos and Leukos within 24 h from the injection. (Hoshyar N 2016). Thus, we can expect that both Lipos and Leukos would have already reached their target tissue or have been removed by filtering organs before they release DOXO into the systemic circulation, avoiding substantial drug leakage. Ultimately, these studies demonstrate how DOXO can be efficiently loaded within our new biomimetic nanovesicle formulations and is released following a pH-dependent and gradual trend.

### 3.4 Assessment of Lipo and Leuko Interaction With Inflamed Endothelial Cells *In Vitro*

The ability of Leukos to adhere to the activated endothelia associated with either local inflammation or tumor development *via* their membrane proteins is an essential feature necessary to enable their efficient active targeting (Zinger et al., 2021). As a straightforward model of endothelial inflammation, HUVEC cells were cultured onto gelatin-coated plates and were either directly incubated with Lipo Cy5.5 or Leuko Cy5.5 or treated with 100 ng/ml of LPS (a component of bacterial walls) to induce their inflammation.

**FIGURE 6:**
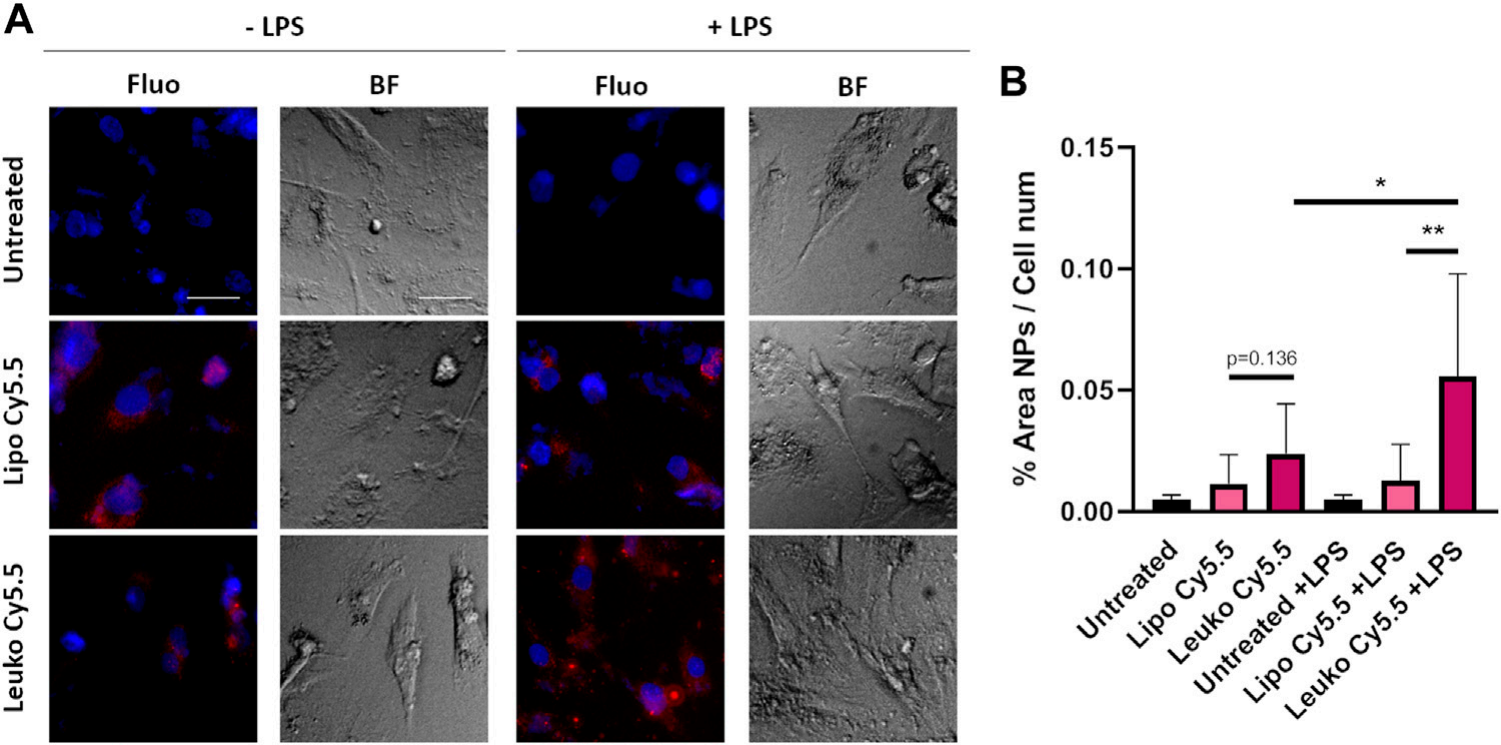
**(A)** Fluorescence (Fluo) and respective bright field (BF) images of HUVEC cells cultured onto gelatin-coated plates and treated with either Lipo Cy5.5 or Leuko Cy5.5 in the presence of 100 ng/ml of LPS or not, for 3 h. **(B)** Quantification of Lipo and Leuko fluorescence at different time points during uptake. (∗*p* ˂ 0.05; ∗∗*p* ˂ 0.01, and *n* = 3 for all the performed experiments).

As shown in Figure 6A and its respective quantification in Figure 6B, the interaction of Lipos-Cy5.5 and Leukos-Cy5.5 was visible to some extent even in HUVEC cells that were not exposed to LPS. In this negative control, Leukos-Cy5.5 still adhered to HUVEC cells slightly more than Lipos-Cy5.5, although their difference was not significant (*p* = 0.136). However, when HUVEC cells were pre-treated with LPS, the uptake of Lipos-Cy5.5 was not significantly increased, while Leukos-Cy5.5 uptake almost doubled on average, becoming significantly higher. This simple study demonstrates, in accordance with previous evidence (Molinaro et al., 2016, E. M. Molinaro R, Design and Development of Biomimetic Nanovesicles Using a Microfluidic Approach. 2018), that Leukos are indeed able to interact more efficiently with inflamed endothelia, providing active targeting. Since the only difference between Lipos and Leukos is represented by the presence of membrane proteins on the latter, the higher adhesion of Leukos to HUVEC can be attributed to these surface proteins, although further investigation is required to elucidate which proteins are responsible for the actual NPs adhesion. A better understanding could prompt the enrichment of said proteins onto Leukos to make them more specific.

### 3.5 Assessment of Uptake by Differentiated Macrophage-Like THP-1 Cells

The presence of immune cells such as macrophages in the tumor milieu is an important factor in determining tumor growth and progression and determining the efficacy of treatment (Zhou et al., 2020; Wang, 2021). Indeed, local and recruited macrophages can both secrete growth factors to sustain tumor growth or can work as “decoys,” absorbing drugs and especially particles and large molecules *via* their very efficient phagocytosis, hindering their possible interactions with target tumor cells. Furthermore, in many inflammatory pathologies, macrophages are recruited to the affected tissue to remove potential external bodies and then sustain tissue remodeling and regeneration. To create a straightforward model of macrophages, THP-1 cells were stimulated with 100 ng/ml of PMA to induce their differentiation into macrophages.

**FIGURE 7:**
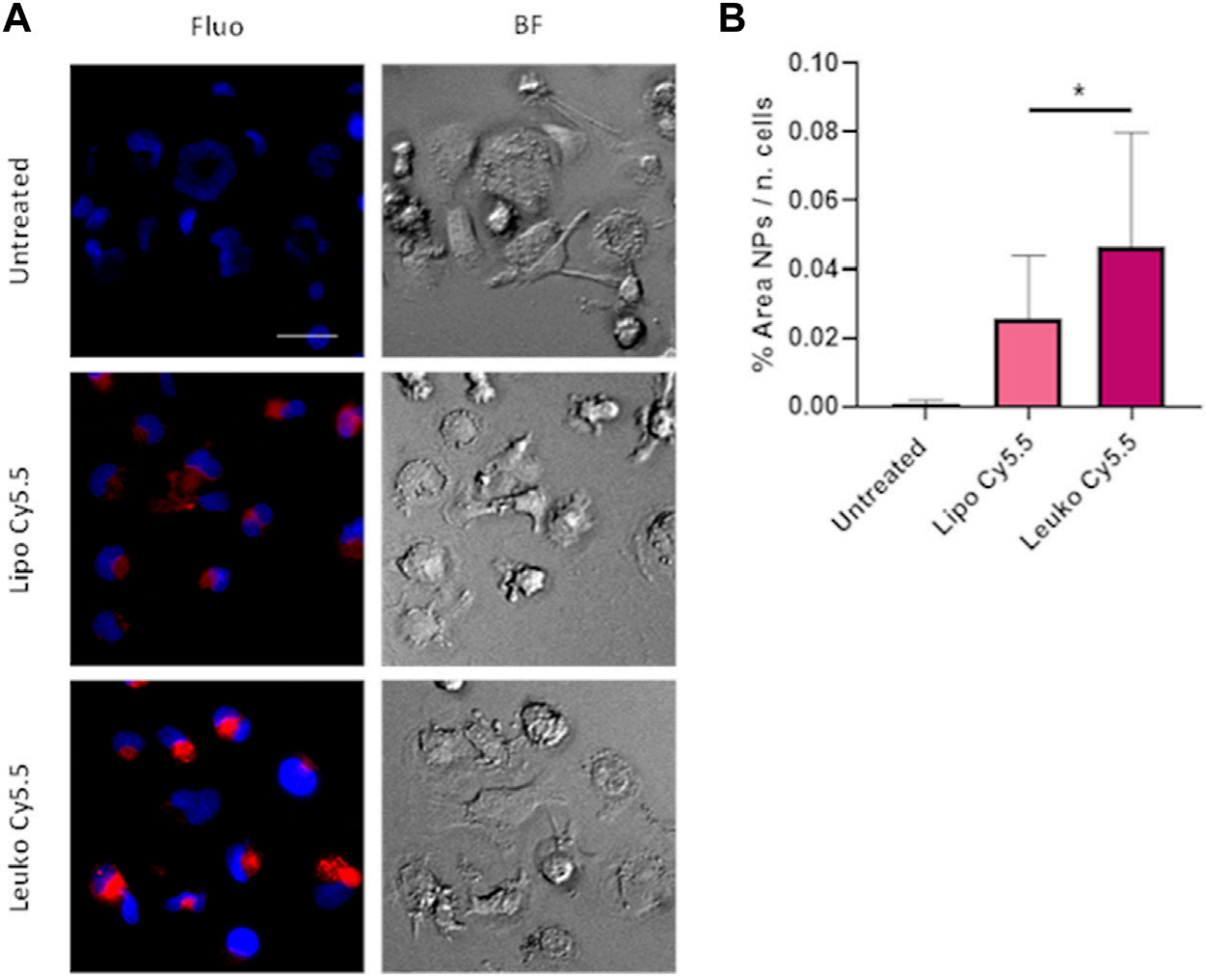
**(A)** Fluorescence (Fluo) and respective bright field (BF) images of THP-1 derived macrophages treated with either Lipo Cy5.5 or Leuko Cy5.5 for 3 h after treatment with 100 ng/ml of PMA for 48 h. **(B)** Quantification of Lipo and Leuko fluorescence at different time points during uptake. (∗*p*˂0.05 and *n* = 3 for all the performed experiments).

As shown in Figure 7A, NPs signal was visible even at the early time point of 3 h, evidencing the efficiency of NPs uptake by differentiated THP-1 cells. The uptake quantification presented in Figure 7B evidence how Leukos-Cy5.5 were internalized more efficiently than Lipos-Cy5.5. It is important to consider that Leukos were formulated using THP-1 derived membrane proteins, and thus Leukos could undergo internalization by activated THP-1 through homologous uptake compared to bare Lipos. As presented in Supplementary Figure S8B, Lipos and Leukos were still well tolerated by differentiated THP-1 at very high concentrations. However, it is also possible that by internalizing Leukos, macrophages can create a drug depot in proximity to the inflamed tissue or tumor, increasing the retention of the particles in the target tissue. This is of great relevance since leukosomes represent prime drug delivery vectors candidates for anti-inflammatory drugs. Leukosomes tropism toward macrophages present in inflamed sites thus represents a second level of targeting specificity beyond endothelial adhesion, which could further improve their efficacy in the treatment of inflammatory pathologies. These observations pave the way for future studies performed by loading corticosteroids within Leukos through an established remote loading strategy (Al-Amin et al., 2020).

Nevertheless, it is important to consider the potential off-target toxicity that Leukos could exert on macrophages and endothelial present across the organism, which could result in systemic adverse reactions. This concern for systemic toxicity was addressed in different studies both using empty leukosomes (Zinger et al., 2021; Molinaro et al., 2016) and leukosomes loaded with DOXO (Molinaro et al., 2020), in which no evident organ or systemic effect was observed in in vivo models of local inflammation (Zinger et al., 2021), breast cancer, and melanoma (Molinaro et al., 2020).

### 3.6 *In Vitro* Assessment of Lipo and Leuko Uptake by Tumor Cells

The assessment of Leukos internalization by tumor cells is paramount since the endocytic uptake of Leukos is necessary to ensure the efficient internalization of the loaded DOXO cargo, leading to its improved efficacy compared to the free drug.

**FIGURE 8:**
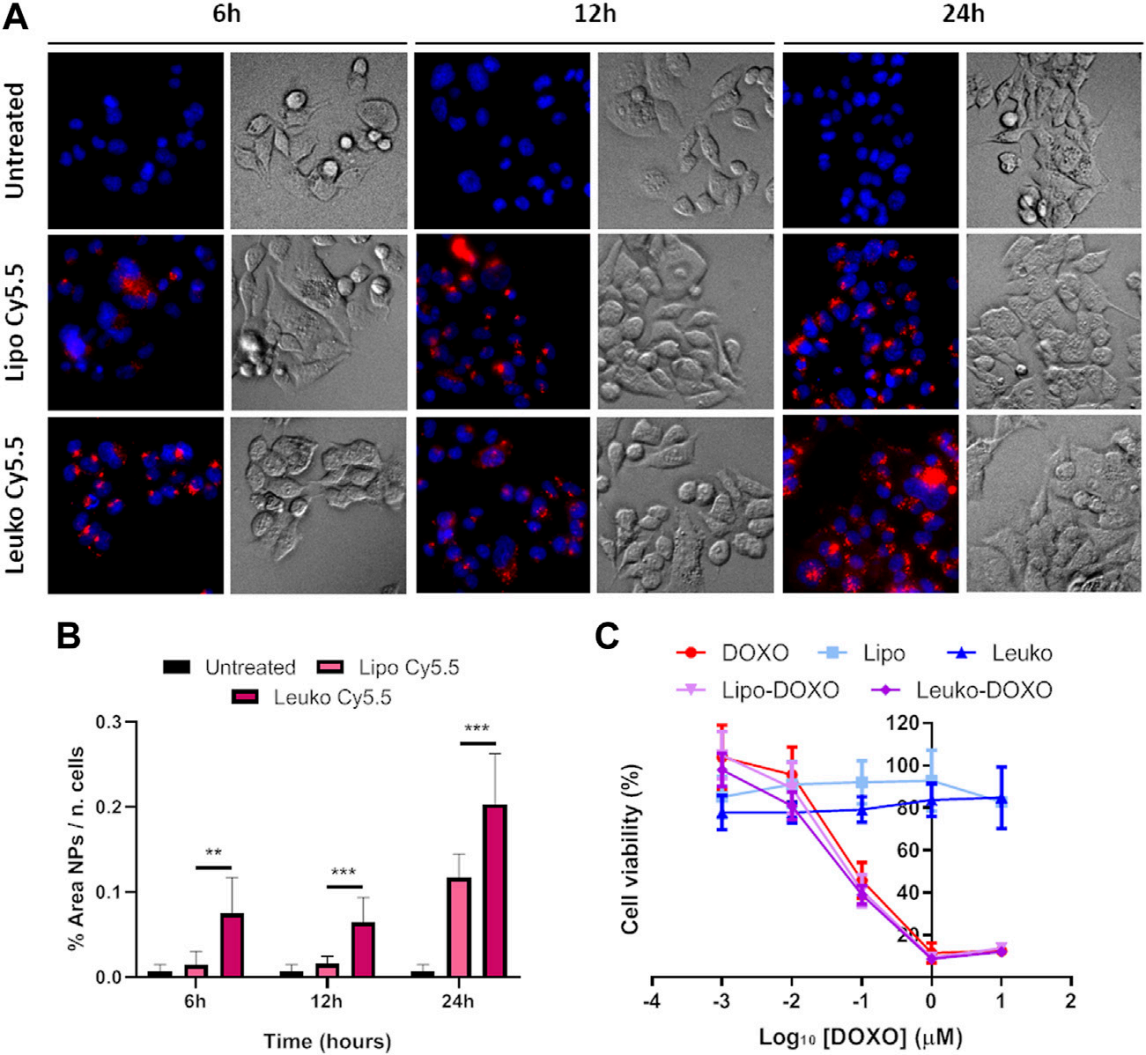
**(A)** Fluorescence (Fluo) and respective bright field (BF) images of HCT-116 cells cultured onto flat plates treated with either Lipo Cy5.5 or Leuko Cy5.5 for 6, 12, and 24 h. **(B)** Quantification of Lipo and Leuko fluorescence at different time points during uptake. **(C)** Resazurin cell viability assay results for DOXO, Lipo, Leuko, Lipo-DOXO, and Leuko-DOXO on HCT-116 cells. (∗∗*p* ˂ 0.01; ∗∗∗*p* ˂ 0.001, and *n* = 3 for all the performed experiments).

Cy5.5 labeled fluorescent Lipos and Leukos (Lipos-Cy5.5. and Leukos-Cy5.5) were used to trace and quantify their uptake rate. As shown in Figure 8A, Lipos-Cy5.5 and Leukos-Cy5.5 were internalized by cells even at 6 h from the initial incubation, as confirmed by the presence of particles within the intracellular space observed in the bright field (BF) channel. Furthermore, the NPs fluorescence in all time points appeared as cytosolic fluorescent spots. Since the size of single particles is too small to be visualized by an optical microscope, it is very likely that Lipos-Cy5.5 and Leukos-Cy5.5 are clustered within some form of intracellular compartment. The most likely hypothesis is that our NPs follow the same fate as many other formulations, and after endocytosis, they are trafficked to the lisosomal compartment. However, the precise identity of this compartment should be defined using *ad hoc* intracellular markers. In addition, the NPs signal tends to concentrate in the perinuclear region, which is very suitable for the released DOXO to exert its cytotoxic effects on the tumor cells’ DNA.

However, it is also important to quantitatively compare the uptake rate of Lipos-Cy5.5 compared to Leukos-Cy5.5 to see if the presence of membrane proteins can influence the kinetics of NPs uptake. To perform this, the NPs fluorescence was measured using the FIJI software and was normalized by dividing it by the number of cells, that was calculated automatically by counting the fluorescent nuclei. As evidenced in Figure 8B, HCT-116 cells demonstrated a significantly higher uptake of Leukos-Cy5.5 compared to Lipos-Cy5.5 at all time points, with both particles’ uptake especially increasing between 12 and 24 h.

However, the use of flat cell cultures gives a somewhat reductive insight into the internalization rates of tumor cells. Indeed, in a flat cell culture setting, all the cells are equally and directly exposed to the treatment since they grow on a flat monolayer. This setup thus does not consider the potential barrier effect provided by the tumor tissue, which can limit drug and NPs diffusion, hindering their interaction with tumor cells. Furthermore, the extracellular matrix is known to have important functions in supporting and even promoting tumor development (Walker et al., 2018; Henke et al., 2020), working as a bioactive scaffold.

**FIGURE 9:**
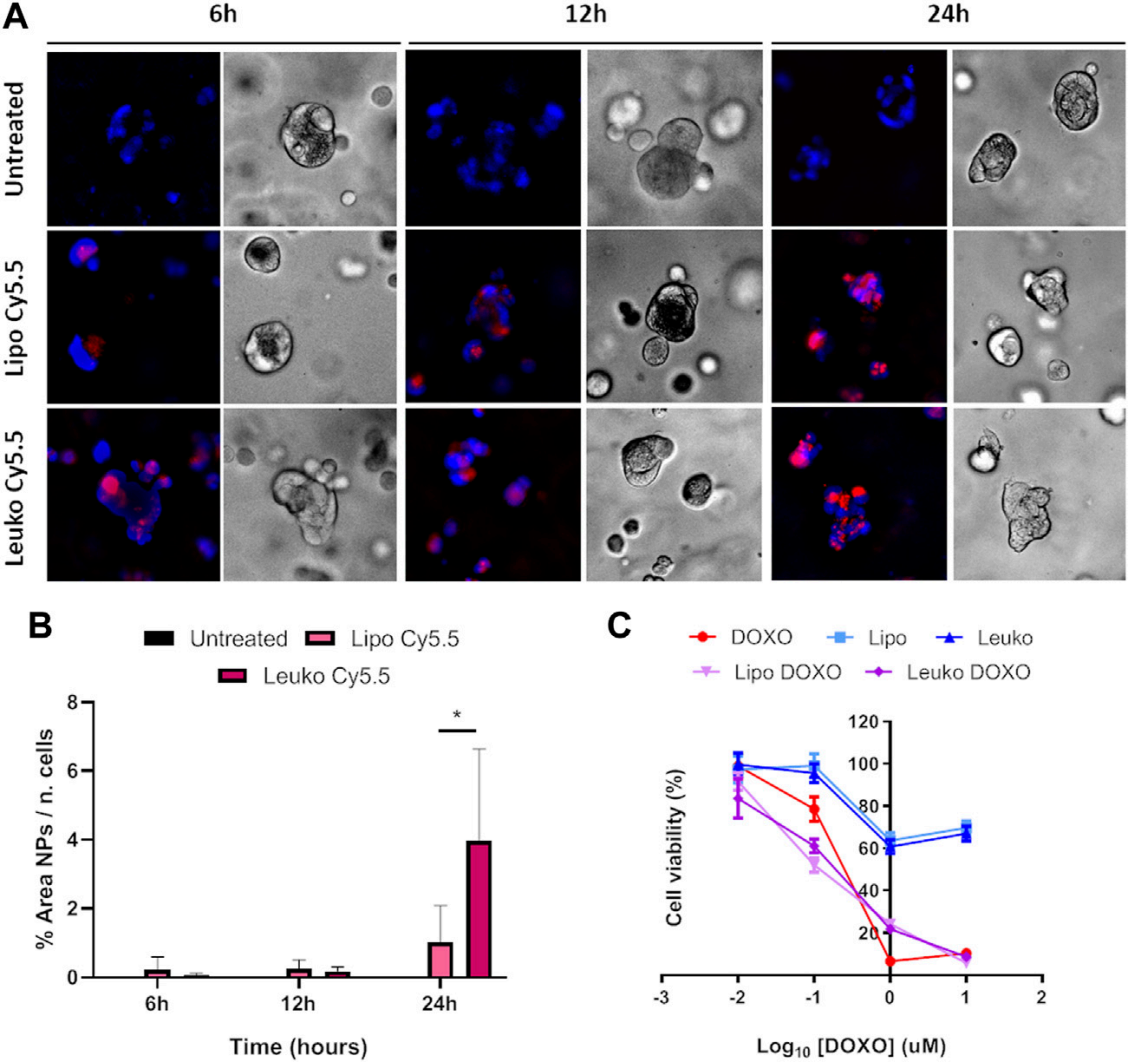
**(A)** Fluorescence (Fluo) and respective bright field (BF) images of HCT-116 spheroids cultured into Geltrex™ treated with either Lipo Cy5.5 or Leuko Cy5.5 for 6, 12, and 24 h. **(B)** Quantification of Lipo and Leuko fluorescence at different time points during uptake. **(C)** Resazurin cell viability assay results for DOXO, Lipo, Leuko, Lipo-DOXO, and Leuko-DOXO on HCT-116 spheroids. (∗*p* ˂ 0.05 and *n* = 3 for all the performed experiments).

To gain further insights into this mechanism, both HCT-116 cells were cultured after being dispersed in a collagen-based, animal-derived hydrogel (Geltrex™). This scaffold has been largely used not only to create a 3D structure to support tumor cells but also to induce cell growth into organoids (Lv et al., 2017). These structures are clusters of tumor cells that more closely mimic solid tumor pathophysiology in terms of cell-cell and cell-matrix contacts and the development of biological gradient stimuli. Specifically, the fact that external cells are more easily exposed to nutrients, oxygen, and the treatment, while internal cells are deprived, can provide important information about the diffusion of treatments into the tumor mass. Furthermore, this peculiar geometry can also modulate the gene expression of tumor cells toward a phenotype that results in higher drug resistance and is associated with more invasive profiles. Thus, 3D cultured HCT-116 cells cultured in Geltrex™ were incubated with 5 × 10^10^ Lipos-Cy5.5 or Leukos-Cy5.5 per ml of complete medium, stained for their nuclei, and imaged at 6, 12, and 24 h. As shown in Figure 9A, tumor cells in Geltrex™ internalized Lipos-Cy5.5 and Leukos-Cy5.5 even at the shortest time point of 6 h, and the NPs fluorescence was visible as spots throughout the entire tumor organoids for both cell lines, demonstrating efficient NPs penetration of the gel. Remarkably, the uptake trend was quite different compared to the respective two-dimensional setup. Specifically, HCT-116 cells demonstrated a marked increase of uptake at 24 h compared to the other time points (Figure 9B) and compared to the 2D setting in which the uptake was more gradual, and the difference in uptake between Lipos and Leukos was more marked (three times different). This was especially visible for Leukos-Cy5.5, which demonstrated a significantly higher uptake than Lipo Cy5.5 at 24 h.

### 3.7 Assessment of Cytotoxicity on Tumor Cells and Patient-Derived Tumor Organoids

The cytotoxic effect of Lipos-DOXO and Leukos-DOXO was also assessed both in 2D and 3D settings. Specifically, HCT-116 cells were seeded onto 96-well plates or in Geltrex in similar conditions to the ones employed for the uptake studies. Then cells were treated with suspensions of either DOXO, Lipos, Leukos, Lipos-DOXO, or Leukos-DOXO dispersed in a complete culture medium and in the concentration range of 0.01–10 µM. Cells were then incubated with the treatment for 72 h, and then their viability was assessed using the Resazurin assay. The resulting IC_50_ and their respective *R* ^2^ values are reported in Table 1.

**TABLE 1:** Summary of the IC50 values for DOXO, empty Lipo, empty Leuko, Lipo-DOXO and Leuko-DOXO.

Conditions	Value	DOXO	Lipo	Leuko	Lipo-DOXO	Leuko-DOXO
HCT-116 2D	IC_50_ (µM)	0.100	NA	NA	0.075	0.060
HCT-116 3D		0.217	NA	NA	0.120	0.185
PDOs		3,584	NA	NA	3,849	1,239

As shown in Figure 8C, DOXO treatment on HCT-116 cells had an IC_50_ value of around 0.1 µM. Conversely, it was not possible to calculate the IC_50_ empty Lipos and Leukos since they appeared to be well tolerated by tumor cells. Interestingly, Lipos-DOXO and Leukos-DOXO were slightly more toxic compared to the free drug since they achieved an IC_50_ of 0.075 and 0.060 µM, respectively, on CRC cells, demonstrating a comparable effect of DOXO when encapsulated in NPs.

However, 3D cultured cell lines treated in analogous conditions presented a different toxicity profile (Figure 9C). Specifically, HCT-116 cells have an IC_50_ for free DOXO that was double of the respective 2D cell culture (0.217 μM). In this case, empty Lipos and Leukos were still well tolerated by the cells, while Lipos-DOXO and Leukos-DOXO demonstrated a similar, albeit slightly decreased IC_50_ (0.120 and 0.185 μM, respectively).

These data underline how particles, despite being very well tolerated by tumor cells in 3D, maintained the antitumor effect of DOXO. It is important to note that the difference in the values of IC_50_ is most likely not caused by a barrier effect provided by the Geltrex™. This is proven by the fact that, in the uptake experiments, both Lipos and Leukos were able to efficiently reach the cells embedded in the hydrogel within 24 h from incubation. Therefore, since the cell viability for these experiments was assessed at 72 h from incubation with NPs, the possible barrier effect provided by the Geltrex™ was overcome. This would make the effects of Lipo and Leuko more comparable since both particles would reach the cells easily and have more time to be internalized. Thus, the difference in the cytotoxic effect of both the free drug is to be attributed to the bioactive stimuli provided by the hydrogel scaffold itself, which supports tumor cells’ growth and metabolism.

### 3.8 *In Vitro* Assessment of Cytotoxicity on Patient-Derived Tumor Organoids

**FIGURE 10:**
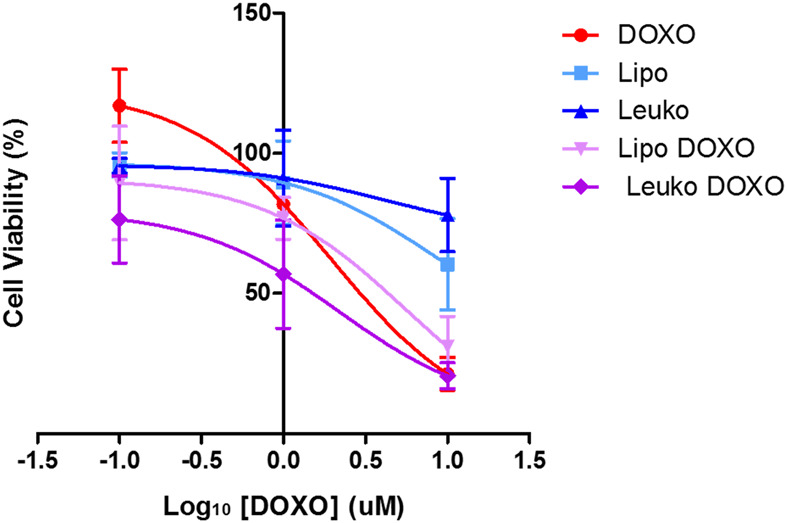
Cytotoxicity effect of DOXO, Lipo, Leuko, Lipo-DOXO, and Leuko-DOXO on PDSs. (*n* = 3).

We tested the cytotoxicity of DOXO, Lipo, Leuko, Lipo-DOXO, and Leuko-DOXO using the same parameters employed for CRC cell lines. Of note, PDOs viability was assessed using ATP quantification through Cell-Titer-Glo assay since Resazurin did not show relevant fluorescence even after a long incubation time (data not shown). This makes it difficult to compare these data with the IC_50_ values obtained from the cell lines. Nevertheless, as presented in Figure 10 and Table 1, the IC_50_ values were 3.584 µM for DOXO, 3.849 µM for Lipo-DOXO, and 1.239 for Leuko-DOXO. As assessed for CRC cell lines, empty Lipo and Leuko do not show relevant toxic effects *per se*. It is interesting to note that Lipo-DOXO had a comparable cytotoxic effect to free DOXO, while Leuko-DOXO toxicity was almost doubled. These data further reinforce the potential of Leuko as a drug delivery system using a highly translational *in vitro* model of CRC. The implementation of these relatively simple models on a larger scale could give a better insight into the efficacy of many NPs formulations that could complement the more established *in vivo* murine models of solid tumors.

Nevertheless, the difference in toxicity between free DOXO and DOXO-loaded particles is not statistically relevant despite the slightly reduced IC_50_ values in cell lines and PDOs. Despite this comparable efficacy, the results derived from the *in vitro* targeting experiments on HUVEC cells (Section 3.4) underline how Leukos have a significant targeting selectivity toward inflamed vessels associated with both inflammatory pathologies and many solid tumors, including colorectal cancer (Schmitt, 2021). This would translate to better Leukos accumulation within the target tissue milieu *in vivo*, resulting in higher drug exposition of tumor cells to DOXO compared to free drug administration (Molinaro et al., 2020). The higher effective DOXO dose created *in loco* could ultimately improve DOXO antitumor efficacy. Like other drug delivery vectors, Leukos would thus work as a device to manipulate the drug pharmacokinetics toward a more favorable biodistribution.

Finally, despite the potential advantage of using NPs to bypass drug resistance in tumor cells (Section 3.2), these proof-of-concept studies are not designed to assess this specific mechanism of action for either Lipos or Leukos. Further studies employing well-known DOXO-resistant cell lines would be warranted.

## 4 Conclusion

In the present work, a novel biomimetic nanovesicle formulation was optimized to be used as a potential drug delivery system to target the inflamed vasculature associated with colorectal cancer. To achieve this, phospholipid nanovesicles have been functionalized with leukocyte-derived membrane proteins to provide active targeting toward the inflamed endothelia *via* adhesion proteins and improve the effect of their cargo, defined as Leukos.

The assembly of nanoparticles was performed in a single step using the microfluidics-based platform Nanoassemblr™. To optimize this process and achieve monodisperse nano-sized Leukos with a high protein loading, a design of experiment approach was employed. Desiring Leukos with a size around 150 nm and a minimized PDI and zeta potential, the model retro-calculated, as necessary experimental parameters, a TFR of 1 ml/min, an FRR of 4.88:1, and a lipid to proteins ratio of 20:1. The predictions were validated by formulating Leuko using the calculated parameters, which demonstrated the correctness of the predictions, with Leuko 150 nm in diameter, a PDI of 0.24, and zeta potential of −25 mV that was significantly lower compared to Liposomes with the same composition but without membrane proteins. Thus, this formulation was selected for further studies. These Leukos were demonstrated to be colloidally stable, maintaining their main features until 15 days from synthesis. Furthermore, this formulation demonstrated the presence and correct orientation of key membrane proteins on its surface as demonstrated *via* flow cytometry, retaining important biomolecules involved in cell adhesion. However, the potential of Leukos as drug delivery systems depends on its ability to efficiently encapsulate therapeutic agents. As a model drug, the antitumor molecule DOXO was encapsulated using a remote loading approach, reaching a maximal encapsulation efficiency of 60% of the incubated drug. Furthermore, the release of DOXO from Leukos was pH-dependent, with the drug being released from the particles faster at a slightly acidic pH (6) compared to physiological pH (7.4), which is ideal to guarantee the release of DOXO only after particles internalization and trafficking to the acidic lysosomal compartment.

We then focused on the assessment of Leukos adhesion to inflamed endothelial cells. Remarkably, Leukos were able to efficiently adhere to HUVEC endothelial cells inflamed with LPS compared to both Lipos and Leukos incubated onto non-inflamed endothelial cells demonstrating selectivity toward inflamed endothelia. We also tested the interaction between Lipos, Leukos, and differentiated THP-1 cells as a model for immune cells responsible for NPs clearance. Leukos were internalized more efficiently compared to Lipos, demonstrating the ability to potentially be retained within the tissue creating a local drug depot. Furthermore, Leukos were also efficiently internalized by HCT-116 cells cultured both in a flat condition and using the collagen-based Geltrex™ hydrogel as a bioactive scaffold to induce tumor organoids. Importantly, Leukos demonstrated a higher uptake compared to bare Lipos. Since the internalized NPs presented a cytosolic, punctate, and perinuclear intracellular accumulation, it is possible to postulate that NPs are internalized *via* some form of endocytic uptake, although more investigation would be warranted to ensure Leukos intracellular trafficking destination. Lipos-DOXO and Leukos-DOXO exerted a similar cytotoxic effect against human HCT-116 colorectal cancer cells compared to free DOXO, while empty NPs did not exert any relevant cytotoxic effect. Furthermore, colon cancer cells cultured in 3D demonstrated more resilience to the treatments compared to their respective two-dimensional cultures, proving the important contribution of the extracellular matrix to cancer growth *via* bioactive functions and not simply *via* a barrier effect that prevents drug uptake. The increased efficacy of Leuko-DOXO compared to Lipo-DOXO was further confirmed by PDOs as an alternative colorectal cancer *in vitro* model.

Nevertheless, these *in vitro* models constitute only preliminary biological tests for our biomimetic nanovesicles and their interactions with and response to Leukos constitute an important preliminary step to confirm Leuko functions in a well-controlled and simplified biological environment. Further *in vivo* studies in the future will provide important information to complement the insights presented in this article. Specifically, *in vivo* studies will elucidate the biodistribution and targeting efficacy of Leukos after intravenous administration to solid tumors and local inflammation models, will evidence their possible off-target accumulation, and assess if Leuko can cause organ toxicity, systemic adverse reactions, or elicit an immunological response from the organism.

In conclusion, this work constitutes a proof of concept for the creation of a novel framework for the formulation, optimization, and characterization of biomimetic NPs. This was performed starting with the assembly of the nanovesicles and their physical and chemical characterization using a DoE strategy for their biological testing on different tumor and tissue models. Hopefully, this workflow can be used in the future for the formulation of biomimetic nanovectors for many other therapeutic applications.

## Data Availability

The raw data supporting the conclusion of this article will be made available by the authors, without undue reservation.

## Abbreviations

BF, bright field; CRC, colorectal cancer; Cy5.5, cyanine 5.5; DoE, design of experiment; DOPC, 1, 2-dioleoyl-sn-glycero-3-phosphocholine; DOXO, doxorubicin; DPPC, 1, 2-dipalmitoyl-sn-glycero-3-phosphocholine; FRR, flow rate ratio; Leuko, leukosomes; Lipo, liposomes; Lip/Prot ratio, lipids to proteins ratio; NPs, nanoparticles; PDI, polydispersity index; PDO, patient-derived organoids; SM, storage medium; TFR, total flow rate.
