# Supplementary material for: Optimization of Biomimetic, Leukocyte-Mimicking Nanovesicles for Drug Delivery Against Colorectal Cancer Using a Design of Experiment Approach
Source: Front Bioeng Biotechnol. 2022 Jun 8;10:883034. doi: 10.3389/fbioe.2022.883034 (PMC9214241; doi:10.3389/fbioe.2022.883034)
Supplement: Supplementary file 1 [file DataSheet1.DOCX]

Supplementary Material


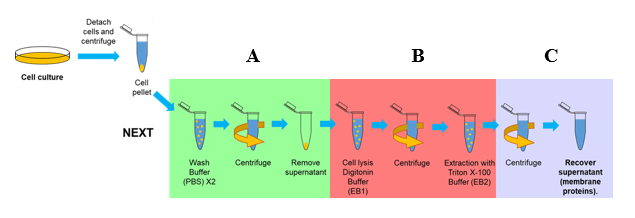


Supplementary Figure 1: Schematic description of the membrane extraction protocol from murine and human monocytes

**Protocol for PDSs extraction:**

| **Storage Medium (SM)** | **Washing Buffer (WB)** | **Digestion Mix (MixD)** | **Culture medium (MS)** |
| --- | --- | --- | --- |
| Primocin (1:500) (InVivoGen, ant-pm-05) | Primocin (1:500) (InVivoGen, ant-pm-05) | Primocin (1:500) (InVivoGen, ant-pm-05) | Primocin (1:500) (InVivoGen, ant-pm-05) |
|  | HEPES 1X (1:100)(Sigma, 83264) | Liberase (50µg/mL) | Growth factors Miltenyi (1:50) (Miltenyi, 130-127-169) |
|  | GlutaMAX 1X (Thermo, 35050061) | ROCK inhibitor 1:1000 (Abcam, ab120129) | ROCK inhibitor 1:1000 (Abcam, ab120129) |
| Amphotericin B (1:100) (Sigma, A2942) | Amphotericin B (1:100) (Sigma, A2942) | Amphotericin B (1:100) (Sigma, A2942) | Amphotericin B (1:100) (Sigma, A2942) |
| Pen/Strep Solution (1:100) (Gibco™ 15140122) | Pen/Strep Solution (1:100) (Gibco™ 15140122) | Pen/Strep Solution (1:100) (Gibco™ 15140122) |  |
| Bring to volume with MACS® Tissue Storage Solution (Miltenyi, 130-100-008) | Bring to volume with DMEM F12 (Gibco) | Bring to volume with DMEM F12 (Gibco) | Bring to volume with Colon TumorMACS™ Medium (Miltenyi, 130-127-169) |

**Supplementary Table 1**: Composition of the different buffers used for organoids extraction. When concentration is not specified, the dilution factor is calculated from the concentration of solutions indicated by the producer.

1. Keep biopsies at 4°C in Storage medium Miltenyi supplemented with Amphotericin 1: 100, PenStrep 1: 100 and Primocine 1: 500 for a maximum of 48 hours.
2. Upon extraction, cool the WB and centrifuge to 4°C. Put Geltrex to thaw on ice.
3. Transfer biopsies to a Falcon tube containing WB and shake gently. Centrifuge the biopsy at 1200rpm for 5 min, 4°C. Remove the supernatant and repeat the wash.
4. Transfer the washed biopsies to a clean glass slide and mince them thoroughly with a scalpel.
5. Transfer the shredded pieces to a 24-wells plate. Add 750-1000uL of MixD per well and incubate to digest for 1h.
6. Every 10 minutes, use a 1000 pipette with cut tips to mix the MixD and the sample.
7. Transfer the digested samples in Falcon tubes containing 8 mL of WB to neutralize the liberase. Use the WB to rinse the wells and recover any remaining debris on the cockpit. Centrifuge at 1200rpm for 5 min, 4°C.
8. Remove supernatant and add 15-20ml of WB and resuspend vigorously with a pipette.
9. Filter the cells using a previously primed Miltenyi Smart Strainer (or 70μm filter) with WB, adding the volume a little at a time.
10. Transfer the cell suspension to one or more Falcon tubes and centrifuge at 1200rpm for 5 min, 4°C.
11. Remove the supernatant. If blood is visible (red ring around or on top of the pellet) add 1-2mL of Lysis Buffer (Thermo, A1049201, gently resuspend and incubate for 5 min at 37°C. Add 10 mL of washing buffer and centrifuge again at 1200rpm for 5 min, 4°C.
12. Resuspend the pellet 2-3mL of WB and count the cells to seed 100K / drop.
13. Prepare 1.5mL Eppendorf tubes by placing a volume equal to a multiple of 100,000 in each

cells and centrifuge at 1200rpm for 5 min, 4°C.

1. Remove the supernatant and resuspend the pellet in 15-20ul of Geltrex^TM^ per 100K cells. Gently place each drop of 15-20ul gel in a pre-heated 48-24 well plate incubator. Invert the plate upside down and place it in incubator for 30 min.
2. Remove the plate from the incubator, check that the gel has solidified, and add gently 250-500ul (for 48 and 24 well plates respectively) of MS per well. Check the appearance of the drops under the microscope.
3. Change the medium every 48h. Based on the confluence of the organoids, perform a split every ½ weeks.


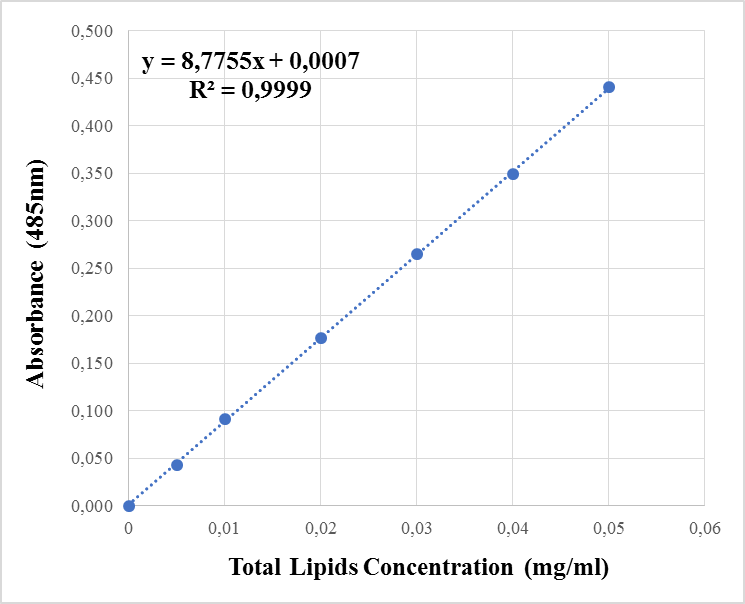


Supplementary Figure 2: Calibration line used for the quantification of Lipo and Leuko Lipids using the Stewart assay. The measurements were performed in triplicate.


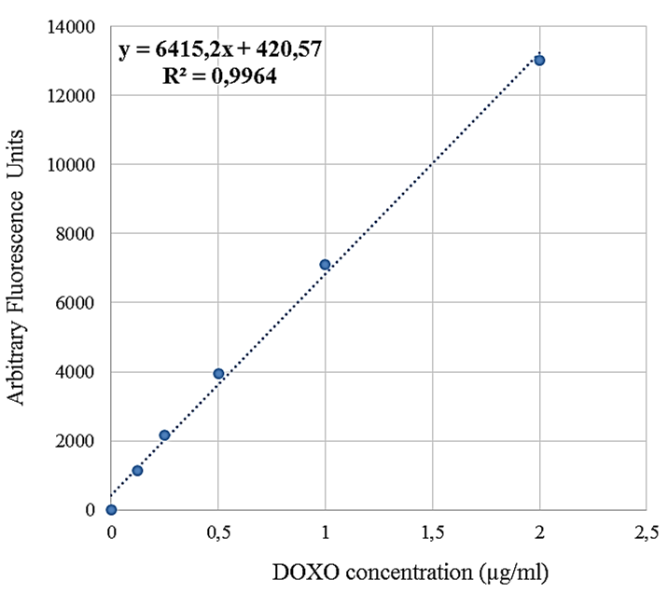


Supplementary Figure 3: Calibration line used for the quantification of DOXO using the fluorimetry. The measurements were performed in triplicate.


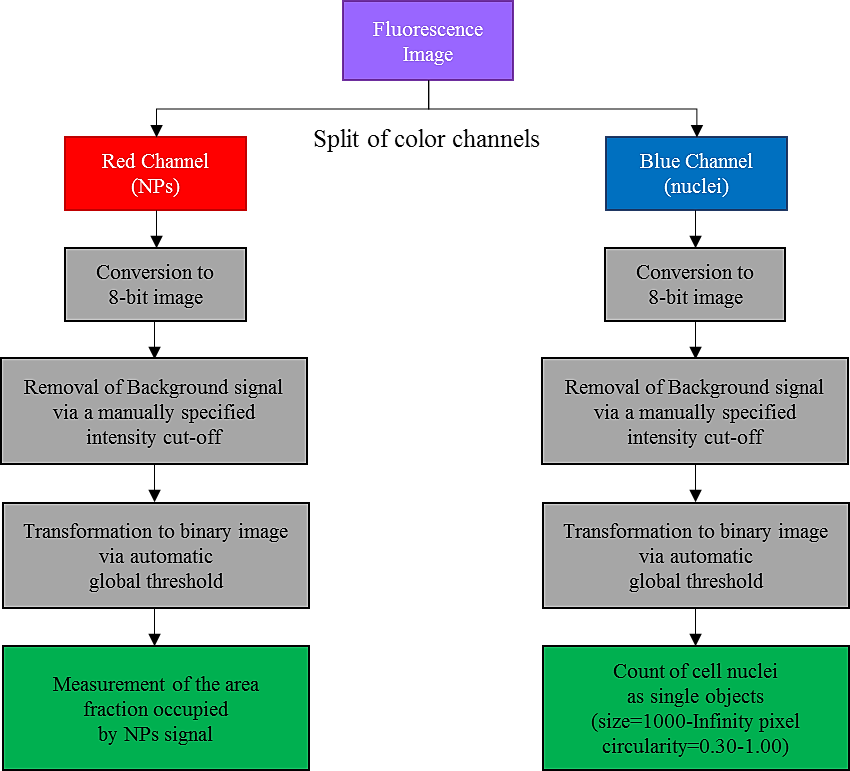


Supplementary Figure 4: schematic representation of the workflow used for the processing of fluorescence images of tumor cells and spheroids to perform NPs uptake quantification.

| Experimental Run | TFR (ml/min) | FRR (acq/org) | Lip/Prot Ratio |
| --- | --- | --- | --- |
| 1 | 5.5 | 3 | 160 |
| 2 | 1 | 3 | 20 |
| 3 | 5.5 | 1 | 300 |
| 4 | 1 | 3 | 300 |
| 5 | 10 | 5 | 160 |
| 6 | 1 | 5 | 160 |
| 7 | 10 | 1 | 160 |
| 8 | 5.5 | 5 | 300 |
| 9 | 1 | 1 | 160 |
| 10 | 10 | 3 | 20 |
| 11 | 5.5 | 5 | 20 |
| 12 | 10 | 3 | 300 |
| 13 | 5.5 | 1 | 20 |

Supplementary Table 2: Different experimental parameters combinations generated to perform a single run of DoE for Leuko optimization.


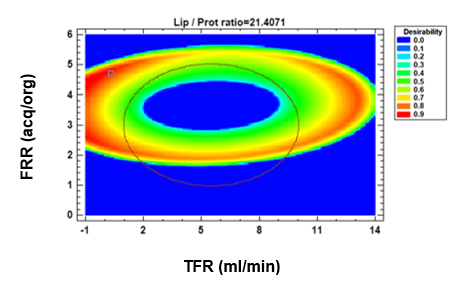


Supplementary Figure 5: Desirability plot calculated from the DoE results and after the selection of ideal Leuko physiochemical features.


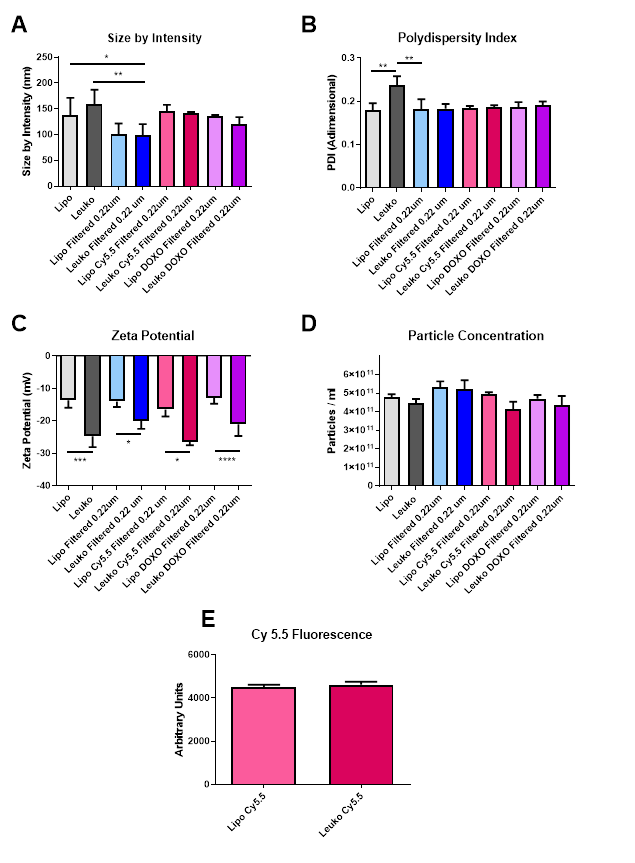


Supplementary Figure 6: fluorescence of Lipo Cy5.5 and Leuko Cy5.5 measured by plate reader after dilution (n=3).


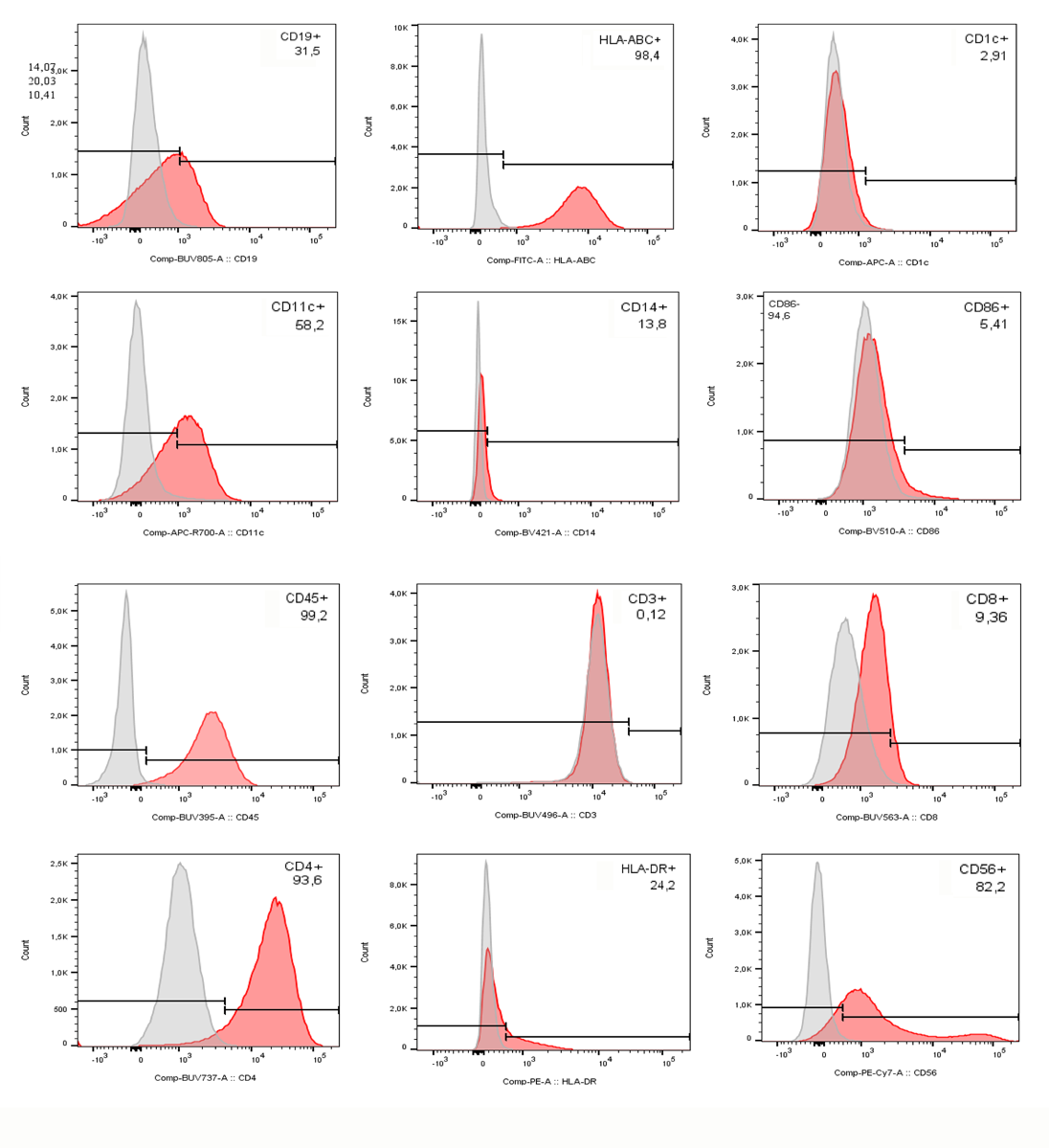


Supplementary Figure 7: Characterization of THP-1 cells via multicolor immune detection. Representative histograms of the expression of different immune cell markers in THP-1 cells (grey represents the negative controls, and red represents the antigen tested). Expression of surface markers was expressed as % MFI. Three independent experiments were performed with similar results.

Supplementary Table 3: Comparison between immune surface markers detected onto THP-1 cells and Leuko by cytometric analysis. The abundance of immune cell antigens both on cells and Leukos membranes was evaluated. The antigens expression levels were divided based on the MFI (median fluorescence intensity) value for THP1 cells in negative: (-) <50, low (+) 50-400, moderate (++) 400-1000, and high (+++) 1000-7000 and for Leukos: (-) <1, (+) 20-110, (++) 110-485, and (+++) 485-800, respectively.


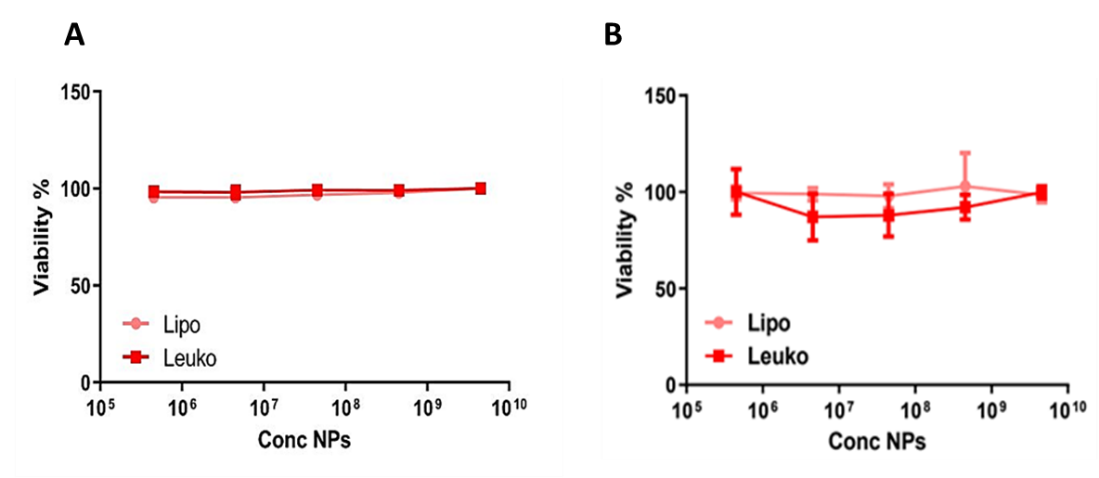


Supplementary Figure 8: Assessment of cell viability.
